# Exploring metapopulation‐scale suppression alternatives for a global invader in a river network experiencing climate change

**DOI:** 10.1111/cobi.13993

**Published:** 2022-12-30

**Authors:** Brian D. Healy, Phaedra Budy, Charles. B. Yackulic, Brendan P. Murphy, Robert C. Schelly, Mark C. McKinstry

**Affiliations:** ^1^ Department of Watershed Sciences and the Ecology Center Utah State University Logan Utah USA; ^2^ Native Fish Ecology and Conservation Program, Division of Science and Resource Management Grand Canyon National Park, National Park Service Flagstaff Arizona USA; ^3^ U.S. Geological Survey, Utah Cooperative Fish and Wildlife Research Unit, Department of Watershed Sciences Utah State University Logan Utah USA; ^4^ U.S. Geological Survey, Southwest Biological Science Center Grand Canyon Monitoring and Research Center Flagstaff Arizona USA; ^5^ School of Environmental Science Simon Fraser University Vancouver British Columbia Canada; ^6^ Upper Colorado Regional Office U.S. Bureau of Reclamation Salt Lake City Utah USA

**Keywords:** conservation, demographic rates, flow‐ecology, introduced species, Lefkovitch matrix, non‐native salmonid, population dynamics, conservación, dinámicas poblacionales, ecología de flujos, especie introducida, matriz de Lefkovitch, salmónido no nativo, tasas demográficas, 保护, 种群统计, Lefkovitch矩阵, 种群动态, 流量生态学, 引入物种, 外来鲑科鱼类

## Abstract

Invasive species can dramatically alter ecosystems, but eradication is difficult, and suppression is expensive once they are established. Uncertainties in the potential for expansion and impacts by an invader can lead to delayed and inadequate suppression, allowing for establishment. Metapopulation viability models can aid in planning strategies to improve responses to invaders and lessen invasive species’ impacts, which may be particularly important under climate change. We used a spatially explicit metapopulation viability model to explore suppression strategies for ecologically damaging invasive brown trout (*Salmo trutta*), established in the Colorado River and a tributary in Grand Canyon National Park. Our goals were to estimate the effectiveness of strategies targeting different life stages and subpopulations within a metapopulation; quantify the effectiveness of a rapid response to a new invasion relative to delaying action until establishment; and estimate whether future hydrology and temperature regimes related to climate change and reservoir management affect metapopulation viability and alter the optimal management response. Our models included scenarios targeting different life stages with spatially varying intensities of electrofishing, redd destruction, incentivized angler harvest, piscicides, and a weir. Quasi‐extinction (QE) was obtainable only with metapopulation‐wide suppression targeting multiple life stages. Brown trout population growth rates were most sensitive to changes in age 0 and large adult mortality. The duration of suppression needed to reach QE for a large established subpopulation was 12 years compared with 4 with a rapid response to a new invasion. Isolated subpopulations were vulnerable to suppression; however, connected tributary subpopulations enhanced metapopulation persistence by serving as climate refuges. Water shortages driving changes in reservoir storage and subsequent warming would cause brown trout declines, but metapopulation QE was achieved only through refocusing and increasing suppression. Our modeling approach improves understanding of invasive brown trout metapopulation dynamics, which could lead to more focused and effective invasive species suppression strategies and, ultimately, maintenance of populations of endemic fishes.

## Resumen

Las especies invasoras pueden alterar dramáticamente un ecosistema, pero erradicarlas es complicado y suprimirlas es costoso una vez que están establecidas. Las incertidumbres en el potencial de expansión y el impacto de un invasor pueden derivar en una supresión retardada e inadecuada que permite el establecimiento. Los modelos de viabilidad meta poblacional pueden auxiliar en la planeación de estrategias para mejorar las respuestas ante especies invasoras y disminuir su impacto, lo cual puede ser particularmente importante ante el cambio climático. Usamos un modelo meta poblacional espacialmente explícito para explorar las estrategias de supresión usadas con la trucha café (*Salmo trutta*), una especie invasora y dañina establecida en el Río Colorado en el Parque Nacional del Gran Cañón. Nuestros objetivos fueron estimar la efectividad de las estrategias enfocadas en diferentes etapas de vida y subpoblaciones dentro de una meta población; cuantificar la efectividad de una respuesta rápida ante una nueva invasión en relación a retardar la acción hasta que ocurra el establecimiento; y estimar si los sistemas térmicos e hidrológicos relacionados con el cambio climático y la gestión de cuencas afectarán la viabilidad meta poblacional y alterarán la respuesta óptima de gestión en el futuro. Nuestros modelos incluyeron escenarios enfocados en diferentes etapas de vida con intensidades espacialmente variables de pesca eléctrica, destrucción de redes, cultivo incentivado de pescadores, piscicidas y un dique. La cuasi extinción (CE) sólo se obtuvo con una supresión a nivel meta poblacional enfocada en múltiples etapas de vida. Las tasas de crecimiento poblacional de la trucha fueron más sensibles a los cambios en edad cero y una gran mortalidad adulta. La duración de la supresión requerida para llegar a la CE para una subpoblación grande establecida fue de doce años en comparación con los cuatro de una respuesta rápida a una nueva invasión. Las subpoblaciones aisladas fueron vulnerables a la supresión; sin embargo, las subpoblaciones conectadas por medio de tributarios incrementaron la persistencia meta poblacional al fungir como refugios climáticos. La escasez de agua, cambios impulsores en el almacenamiento de la cuenca y el calentamiento subsecuente causarían declinaciones de la trucha, pero la CE meta poblacional sólo se logró con el reenfoque e incremento de la supresión. Nuestra estrategia de modelado mejora el entendimiento de las dinámicas meta poblacionales de la trucha café invasora, lo cual podría llevar a estrategias de supresión más enfocadas y efectivas y, finalmente, al mantenimiento de las poblaciones de peces endémicos.

Exploración de alternativas a la supresión a escala meta poblacional de un invasor mundial en una red de ríos que experimenta el cambio climático

## 摘要

入侵物种可以极大程度地改变生态系统, 但一旦它们建立种群, 就很难彻底清除, 且控制其种群的费用也十分高昂。入侵物种潜在的扩张和影响存在不确定性, 这可能造成种群控制行动的延迟和不充分, 从而使其得以建立种群。集合种群生存力模型可以帮助战略规划, 以改进对入侵物种的响应, 减少入侵物种的影响, 这一点在气候变化背景下尤为重要。本研究利用一个空间显式的集合种群生存力模型来探究对破坏生态的入侵物种褐鳟鱼(*Salmo trutta*)的控制策略, 这些褐鳟鱼已在科罗拉多河和大峡谷国家公园的一条支流中建立种群。本研究旨在估计针对集合种群不同生命阶段和亚种群的策略的有效性;量化对新发入侵的快速响应相对于延迟行动至种群建立的有效性;估计与气候变化和水库管理有关的未来水文和温度条件是否会影响集合种群生存力并改变最佳管理响应。我们的模型针对褐鳟鱼不同生命阶段的情景, 包括在空间上进行不同强度的电力捕鱼、破坏鳟鱼产卵区、激励钓鱼者、使用杀鱼剂, 以及建设鱼梁。只有在全流域范围内针对多个生命阶段进行种群控制, 才能实现褐鳟鱼的准灭绝。褐鳟鱼种群增长率对0岁及大型成体死亡率的变化最为敏感。对于一个已建立的大型亚种群, 达到准灭绝所需的控制时间为12年, 而对新发入侵进行快速响应则只需要4年。孤立亚种群容易受到控制;然而, 相互连接的支流亚种群通过作为气候庇护所, 促进了集合种群续存。此外, 水源短缺驱动的水库储量变化及随后的气候变暖会导致褐鳟鱼种群数量下降, 但只有通过重新聚焦并加强种群控制, 才能实现集合种群准灭绝。我们的建模方法增进了对入侵褐鳟鱼集合种群动态的理解, 可以帮助设计更有针对性、更有效的入侵物种控制策略, 最终帮助维持地方特有鱼类的种群。**【翻译:胡怡思;审校:聂永刚】**

## INTRODUCTION

Invasive species can extirpate native species and threaten ecosystem services (Mack et al., 2000; Pyšek et al., 2020); however, eradication of invasive species is difficult and suppression costs increase as populations become established and disperse across the landscape (Simberloff, 2003). Once established, complete eradication is often infeasible due to sociopolitical (Beever et al., 2019) or logistical constraints (Peterson et al., 2008) and costs (Baxter et al., 2008; Mack et al., 2000). Critical uncertainties can also hinder decision‐making and early intervention. The lack of future projections of dispersal or population growth rates, unpredictable extent of ecological or economic damage, and lack of resources needed to control invasive species legitimize inaction (Simberloff, 2003). Consequently, costs may increase, and the likelihood of success declines if suppression is deferred until after populations have fully established and are less vulnerable to stochastic events (Mack et al., 2000; Simberloff, 2003; van Poorten et al., 2019). In addition, socioeconomic beneficiaries (e.g., anglers of introduced salmonids) may resist control of invasive species (Beever et al., 2019; Dunham et al., 2022; Hansen et al., 2019).

Identifying abiotic and biotic drivers of invasive species’ vital rates and planning control operations to target vulnerable or important life stages may improve the effectiveness of suppression strategies (Govindarajulu et al., 2005; van Poorten et al., 2019). This approach may require fundamental but often uncertain knowledge of the species’ population ecology and life history (Simberloff, 2003). Knowledge of vital rates provides an advantage because the effectiveness of control or suppression techniques may be life stage or size specific. For example, invasive amphibians have complex life cycles that may include aquatic egg or larval stages, metamorphosis to a juvenile stage, and sometimes a transition to upland adult habitats, all of which vary in vulnerability to removal techniques (Govindarajulu et al., 2005). Fishing gears used to control invasive fishes, such as electro‐fishing or netting, also select for larger (and thus older) individuals (Healy, Moore, et al., 2022; Koel et al., 2020). Species with complex life histories, including a partial or fully migratory stage, may also require a landscape‐scale approach that controls explicitly for dispersal between populations (Day et al., 2018; Milt et al., 2018).

Metapopulation management approaches are more often applied to imperiled species than to invasive or established non‐native species (Bertolino et al., 2020; With, 2002). Nonetheless, dispersal between populations across spatially heterogeneous landscapes may have important implications for resiliency of suppressed invasive species populations (Day et al., 2018; Pepin et al., 2019; With, 2002). Treating specific locations to eradicate or suppress an open and connected metapopulation of invasive species without a strategic approach, which is common, can lead to failure (Hock et al., 2016; Mack et al., 2000).

Combining matrix‐based projection modeling and population viability analysis (PVA) (Morris & Doak, 2002) can be an effective approach for exploring drivers of population dynamics and the effects of management actions (e.g., Cahn et al., 2011; Kareiva et al., 2000) across a metapopulation (Murphy et al., 2020). A PVA can be used in invasive species management if the aim is to account for suppression varying across life stages of invasive species, meet a minimum population thresholds, or predict and compare the relative likelihood of suppression scenarios leading to eradication and time to extinction (Berg, 2012; van Poorten et al., 2019). Metapopulation‐structured PVA models (mPVAs) are rarely applied to aquatic invasive species, which is surprising given the need to account for dispersal and connectivity between habitats in river networks (Murphy et al., 2020).

Future climate‐driven changes in thermal or flow regimes propagating across dendritic stream networks may facilitate invasions of some, but hinder those of other aquatic species (Rahel & Olden, 2008; Wenger et al., 2011). Recent research involving temperature‐sensitive fishes suggests interconnected tributary and mainstem habitats may provide a diversity of seasonal thermal regimes or complex habitats serving as refuge from disturbance, thereby facilitating persistence of salmonids (Armstrong et al., 2021; Tsuboi et al., 2022). Thus, a need exists to employ spatially explicit mPVAs that incorporate spatial and temporal heterogeneity in habitat and connectivity when evaluating suppression scenarios for invasive aquatic species (Murphy et al., 2020).

We investigated population vulnerabilities to inform suppression strategies for a worldwide, ecologically damaging, invasive salmonid, brown trout (*Salmo trutta*) (Budy et al., 2013; Hansen et al., 2019; McIntosh et al., 2011). Brown trout have been introduced globally and are one of several invasive species responsible for widespread homogenization of fish diversity (Hansen et al., 2019; Toussaint et al., 2016). Where introduced, brown trout generally thrive due to their broad ecological niche, superior competitive abilities, and attainment of large size through predation on abundant naïve prey fishes (Budy et al., 2013; McIntosh et al., 2011). Given the diversity of life‐history strategies exhibited and the ability of populations to quickly rebound due to density‐dependent compensatory survival of young age classes, salmonid suppression programs have often required long‐term multifaceted approaches (Buktenica et al., 2013; Hansen et al., 2019; Koel et al., 2020). Brown trout mechanical suppression has rarely been effective (Caudron & Champigneulle, 2011; Saunders et al., 2015) and then only in small streams and sometimes at great expense over multiple years (Budy et al., 2021; Healy et al., 2020). For instance, in 4 small, isolated streams, 10 electrofishing eradication efforts over 2.5 years were required for success (Carosi et al., 2020).

We explored a range of planned and hypothetical brown trout suppression scenarios, including those targeting different life stages, in the context of a newly established metapopulation threatening native fishes in Grand Canyon National Park (GCNP) to understand the effectiveness of management strategies targeting different life stages and locations within a metapopulation; quantify the effectiveness of a rapid response to a new invasion relative to delaying suppression until establishment; and estimate whether future changes in hydrology and water temperature related to climate change and reservoir management affect metapopulation viability, requiring adaptation of suppression strategies. Given the ubiquitous introductions of salmonids and their worldwide potential to affect aquatic ecosystems and their inhabitants, our results could have far‐reaching implications.

## METHODS

### Study area

Control of brown trout established in GCNP is a priority to mitigate threats of predation to imperiled native fishes in the Colorado River (CR) and its tributaries (Healy et al., 2020; Yard et al., 2011). The National Park Service (NPS) mandates removal of invasive species, where feasible, when natural or cultural resources are negatively affected (U.S. Department of the Interior, 2006). Regardless of the fact that invasive brown trout negatively affect native fishes (Healy et al., 2020), some Indigenous peoples consider native and invasive aquatic life in GCNP culturally important (Runge et al., 2018), and brown trout are a recreationally and economically important species prized by anglers (Beever et al., 2019; Hansen et al., 2019).

**FIGURE 1:**
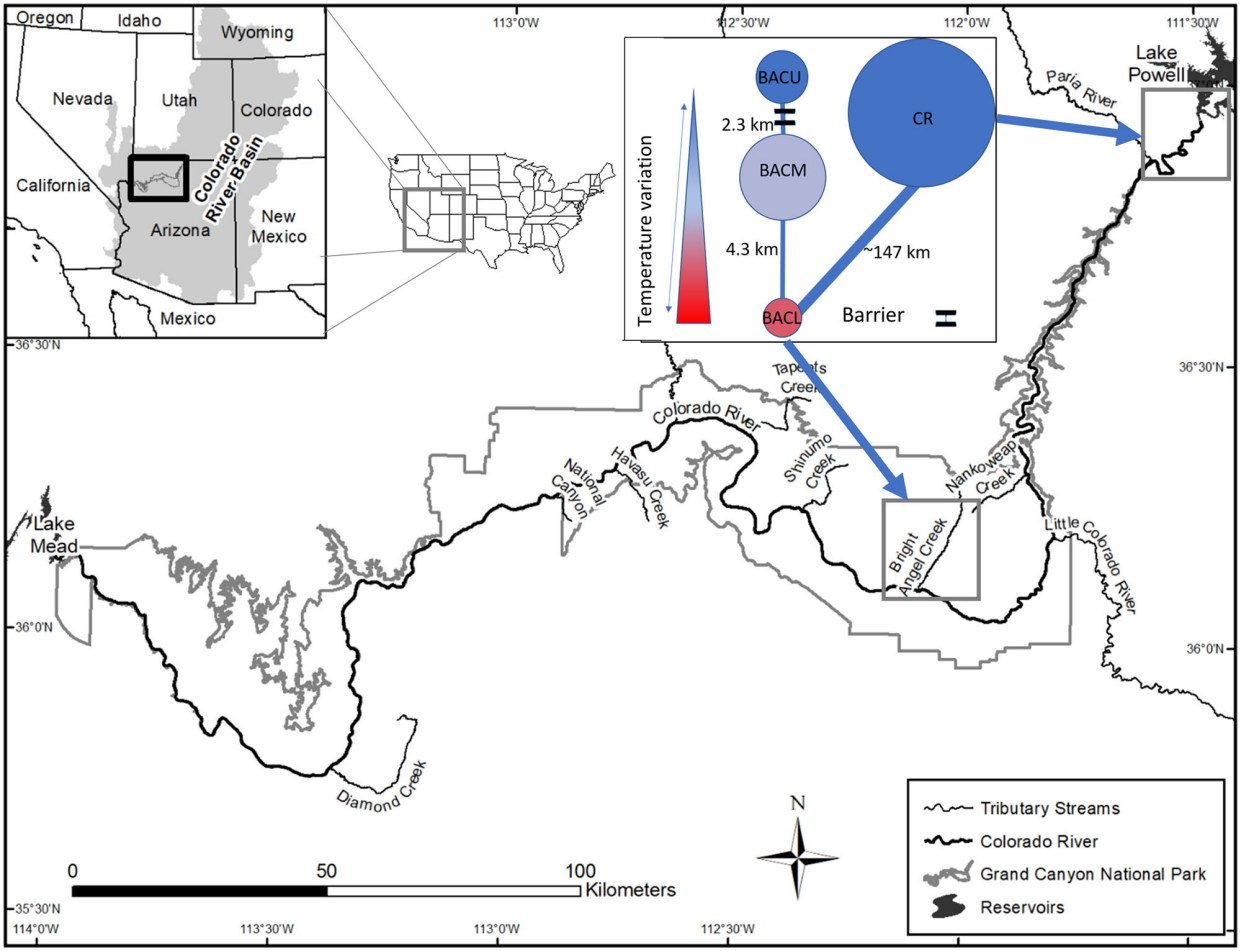
Study area showing the boundaries of Grand Canyon National Park in the Colorado River basin in the southwestern United States. Color inset shows a conceptual metapopulation model including the location of and dispersal distances between each subpopulation in Bright Angel Creek (BAC), including the lower (BACL), middle (BACM), and upper reaches (BACU) and the Colorado River (CR) downstream of Lake Powell and Glen Canyon Dam (circle size, relative carrying capacity for each subpopulation; colors, temperature variation [see scale in inset])

Brown trout were introduced into GCNP tributaries through stocking from the 1920s through the 1930s, and have persisted primarily in 1 tributary, Bright Angel Creek (BAC). The species recently expanded ∼147 km upstream through the CR into the Glen Canyon Dam tailwater, where a second reproducing subpopulation became established (Runge et al., 2018) (Figure 1). Colonization of the tailwater from GCNP was likely facilitated by fall high flow experiments beginning in 2013 (Healy, Yackulic, et al., 2022; Runge et al., 2018); movement in salmonids is commonly stimulated by flow (Davis et al., 2015).

Bright Angel Creek is a perennial spring‐fed stream with a seasonally and longitudinally varying thermal regime (hereafter, temperature) (Figure 1). Temperature is more stable and colder nearest the spring sources (∼11°C, ∼17 km from the mouth), and increasing seasonal variability and warmer summer temperatures characterize downstream reaches (2–25°C) (Bair et al., 2019). Elevated spring snowmelt runoff and monsoon‐driven flooding during summers occur in most years (Healy et al., 2020).

Closure of the Glen Canyon Dam in 1963 and the creation of Lake Powell wrought profound temperature, flow, and sediment regime changes in the CR conducive to trout (Schmidt et al., 1998). Temperature in the mainstem CR is closely linked to water storage in Lake Powell, discharge volume, and air temperature, which in turn may influence fish population status (Dibble et al., 2021). Due to drought and aridification (Udall & Overpeck, 2017), CR temperatures have warmed (2012–2020 range: 7–16°C; U.S. Geological Survey [USGS Gaging Station 09380000] [USGS, 2022]) as reservoir storage has declined (Dibble et al., 2021), and these trends are expected to continue (Wheeler et al., 2021). Decisions regarding future reservoir water storage may lead to even more dramatic variation in temperatures (Dibble et al., 2021). At the same time, climate change is expected to warm temperatures and modify flow regimes in the unregulated tributaries in the Grand Canyon region (Tillman et al., 2020).

### Population viability model

We used a matrix‐based, stage‐structured, spatially explicit, stochastic, partially mechanistic mPVA, the Dynamic Habitat Disturbance and Ecological Resilience Model (DyHDER) (Murphy et al., 2020), to assess suppression strategies and brown trout metapopulation dynamics. The DyHDER was developed specifically to simulate disturbances that may differentially affect dynamics of subpopulations across a landscape while accounting for dispersal and connectively (Murphy et al., 2020) (Appendix S1). The DyHDER model is ideal for simulating management scenarios in the context of future conditions brought about by press disturbances, such as climate change, changes in reservoir storage (hereafter, climate change), and drought that may affect thermal and hydrologic regimes (Dibble et al., 2021; Tillman et al., 2020) and drive brown trout population dynamics (e.g., Lobón‐Cerviá et al., 2018).

Our modeled brown trout metapopulation included 4 subpopulations, including upper (BACU), middle (BACM), and lower (BACL) reaches of BAC and the CR between Glen Canyon Dam and the Paria River (Figure 1). All sites are connected, except the BACU subpopulation, which is upstream of a waterfall impassable to upstream movement of fish, and dispersal to the site was accordingly restricted in the model. We defined 4 life stages of brown trout (age 0, juvenile, small adult, and large adult) and assigned subpopulation carrying capacities (*K*) based on baseline abundance estimates from suppression activities in BAC (Healy et al., 2020). We assumed the most recent abundance estimates approximated *K* for the CR (Appendix S2). We used a combination of empirically or literature‐derived stage‐specific fecundity and demographic and dispersal rate estimates for invasive lotic brown trout populations (Appendix S2).

We incorporated habitat optimality curves (Murphy et al., 2020) in brown trout stage‐transition rates. To account for observed spatial and simulated temporal temperature variation (BAC from Bair et al. [2019]; CR from USGS Gaging Station 09380000 [USGS, 2022]) potentially constraining growth in salmonids (Railsback & Rose, 1999), we applied a temperature optimality curve to transition rates, with temporally varying maximum observed summer daily mean temperatures for each subpopulation and scenario (Appendix S3). Fishes seek temperatures to maximize growth (Hughes & Grand, 2000); thus, we parameterized dispersal as a function of temperature.

Survival of fry may be particularly sensitive to extreme flow events (Lobón‐Cerviá et al., 2018) and warm temperatures (Jonsson, & Jonsson, 2009). We used linear mixed‐effects models to assess relationships between age 0 (*S*_age0_) brown trout abundance, based on data collected from 2012 to 2019 (sampling described in Healy et al. [2020]), and flow and temperature variation for optimality survival curve development. In our candidate models, we included covariates representing temperature and flow volume (mean monthly or seasonal discharge) and flow variability (coefficient of variation of monthly or seasonal discharge) during the winter egg incubation period, spring and summer emergence and growth periods for age 0 fish, and abundance of a potential predator or competitor (age‐1 and older rainbow trout) (Appendix S3). We used Akaike's information criterion (AIC_c_) to compare models, considering models within ΔAIC_c_ = 2 of the top model to be equally supported (Burnham & Anderson, 2002), and converted fitted relationships from the top model to optimality curves for age 0 survival.

**FIGURE 2:**
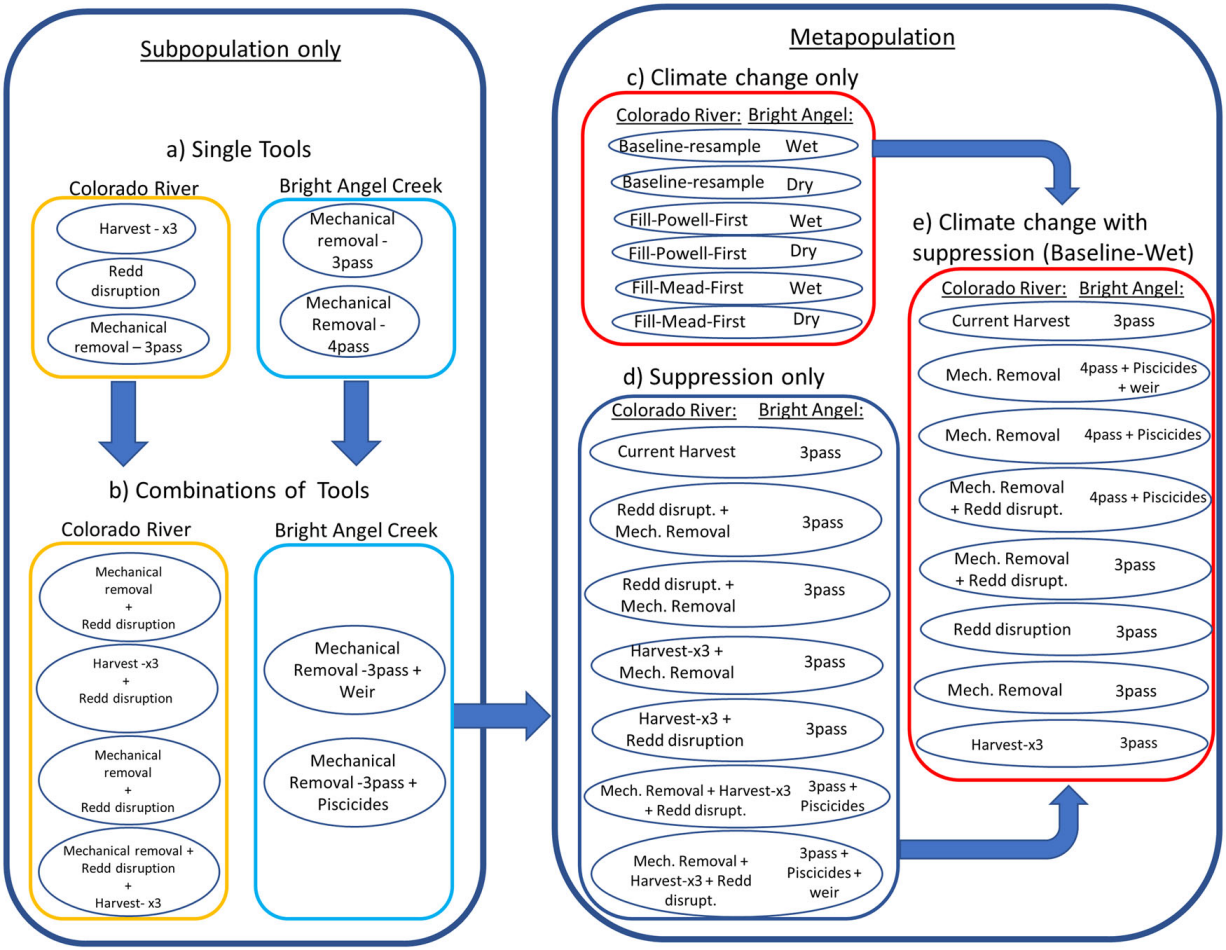
Composition of metapopulation viability model (mPVA) simulation scenarios with suppression or climate change effects for the Colorado River or Bright Angel Creek brown trout subpopulation (left) and metapopulation extents (right). Ovals represent each of 32 scenarios, with arrows depicting the relationship between single subpopulation suppression tools (a) combined into multitool suppression approaches (b), which were then combined into metapopulation‐extent multitool approaches (d). Climate‐change‐effect scenarios without suppression applied across the metapopulation were evaluated (c), and the baseline‐resample‐wet scenario was then combined with metapopulation‐extent suppression scenarios (d), as displayed in (e). (Harvest‐×3, 3 times level of harvest of current level of incentivized harvest; 3‐pass/4‐pass, 3 or 4 passes of mechanical removal electrofishing applied to Bright Angel Creek [only 3 passes applied to the Colorado River and 4 passes applied only across all Bright Angel Creek subpopulations as a single tool or to the BACM subpopulation in panel e]). Details in Appendix S2. Colorado River rapid response suppression scenarios not displayed

We simulated 30‐year brown trout suppression scenarios targeting different life stages across a range of intensity levels, including hypothetical and ongoing management actions, a stable baseline (no suppression), and climate change with and without suppression (Figure 2; Appendix S3). We modeled single‐ and combination‐tool suppression scenarios in CR and BAC separately while maintaining baseline conditions in the nonsuppressed subpopulation and then applied multiple combined suppression approaches to all subpopulations concurrently to assess the importance of dispersal to metapopulation resiliency (Table 1; Figure 2).

**TABLE 1:** Description of brown trout suppression scenarios (including code for each scenario included in Figures 4 & 5), intensity levels of suppression actions, and minimum metapopulation or subpopulation abundance (*N*_min_) over 30 years and time to quasi‐extinction (QE) for each scenario

Code	Scenario description	Meta population *N*_min_	BACL *N*_min_	BACM *N*_min_	BACU *N*_min_	CR *N*_min_	QE time
	Baseline/stable subpopulations at carrying capacity (*K*)	37,529	3811	4305	2971	26,317	–
Colorado River suppression scenarios							
CR‐Harvest	Incentivized angler harvest	19,334	1117	3410	2998	11,772	–
CR‐Redd.Disrupt	Redd disruption	19,162	1127	3398	2995	11,514	–
CR‐Mech.Removal	Mechanical removal – boat‐based electrofishing (3‐pass)	8240	482	3201	2977	1483	–
CR‐Harv.+Redd.Dis	Incentivized harvest + redd disruption	9180	494	3214	2989	2317	–
CR‐Redd.Dis.+Mech.Rem	Redd disruption + mechanical removal (3‐pass)	6679	454	3172	2974	12	–
CR‐Harvest+Mech.Rem.	Incentivized harvest + mechanical removal (3‐pass)	6669	454	3169	2990	12	–
CR‐All.methods	Incentivized harvest + redd disruption+ mechanical removal (3‐pass)	6647	459	3193	2984	4	–
Bright Angel Creek Suppression scenarios							
BAC‐3pass+Weir	Mechanical removal (3‐pass electrofishing) with weir installation	40,548	0	0	1	30,000	–
BAC‐3pass	Mechanical removal (3‐pass electrofishing)	27,795	1108	50	1	26,617	–
BAC‐3pass+Pisc	Mechanical removal (3‐pass electrofishing) and piscicide applied to BACU	27,844	1112	49	0	26,647	–
BAC‐4passes	Mechanical removal (4‐pass electrofishing) stream‐wide	27,078	560	8	0	26,510	–
Metapopulation‐scale suppression scenarios							
CR+BAC‐CurrentSuppression	Incentivized harvest at current level and BAC 3‐pass electrofishing stream wide	21,527	671	23	0	20,830	–
CR‐Redd.+Mech.Rem.+BAC‐3pass	CR—Redd disruption and mechanical removal with 3‐pass electrofishing stream wide	0	0	0	0	0	10.0
CR‐Harv.+Mech.+BAC‐3pass	CR—incentivized harvest and mechanical removal with 3‐pass stream wide electrofishing	0	0	0	0	0	10.3
CR‐Harv.+Redd+BAC‐3pass	CR—incentivized harvest and redd disruption with 3‐pass stream wide electrofishing	0	0	0	0	0	27.3
CR‐All+BAC‐3pass	CR—all suppression methods with 3‐pass stream wide electrofishing	0	0	0	0	0	6.3
CR‐All+BAC‐3pass+Pisc	CR—all suppression methods with 3‐pass electrofishing with piscicides applied to BACU	0	0	0	0	0	5.7
CR‐All+BAC‐3pass+Pisc+Weir	CR—all suppression methods with 3‐pass electrofishing with piscicides applied to BACU and weir installation	0	0	0	0	0	11.4
Rapid response scenarios							
RapidRbaseline	CR—baseline unsuppressed growth for comparison to rapid response suppression	8871	549	3247	2974	1608	–
RapidR.3‐pass	CR—Rapid response to small/increasing population using boat electrofishing	8043	478	3155	2991	1303	13
RapidR.3‐pass+Redd	CR—Rapid response to small/increasing population using boat electrofishing and redd disruption	6645	445	3134	2988	65	4
RapidK.3‐pass+Redd	CR—boat electrofishing and redd disruption applied to stable subpopulation at carrying capacity	6708	463	3198	2996	12	12
Climate change scenarios							
ClimateCRbasewet	Baseline2000 resample with annual max (based on means of 100 traces), BAC RCP 4.5 SWE and Tillman temp increases, with wetter model	10,771	374	1575	1180	7505	–
ClimateCRbasedry	Baseline2000 resample with annual max (based on means of 100 traces), BAC RCP 4.5 SWE and Tillman temp increases, with drier model	9422	335	959	683	7416	–
ClimateCRFPFwet	Baseline2000 resample with FillPowellFirst, BAC RCP 4.5 SWE and Tillman temp increases, with wetter model	12,408	528	1785	1173	8796	–
ClimateCRFPFdry	Baseline2000 resample with FillPowellFirst, BAC RCP 4.5 SWE and Tillman temp increases, with drier model	11,100	491	1152	682	8775	–
ClimateCRFMFwet	Baseline2000 resample with FillMeadFirst, BAC RCP 4.5 SWE and Tillman temp increases, with wetter model	2708	48	1336	1141	0	–
ClimateCRFMFdry	Baseline2000 resample with FillMeadFirst, BAC RCP 4.5 SWE and Tillman temp increases, with drier model	1133	5	407	702	0	16.3
Suppression scenarios with climate change							
Climate.CR+BAC‐Current	Current level of suppression with basewet climate scenario	4550	64	45	0	4439	–
Climate.CR‐3p+BAC‐4p+Pisc+Weir	CR—3‐pass mechanical removal, BACM 4‐pass electrofishing, BACU piscicide application and weir installation	0	0	0	0	0	15.7
Climate.CR‐3p+BAC‐4p+Pisc	CR—3‐pass mechanical removal, BACM 4‐pass electrofishing, BACU piscicide application	0	0	0	0	0	14.5
Climate.CR‐3p+Redd+BAC‐4p+Pisc	CR—3‐pass mechanical removal and redd disruption, BACM 4‐pass electrofishing, BACU piscicide application	0	0	0	0	0	9.0
Climate.CR‐3p+Redd+BAC‐3p	CR—3‐pass mechanical removal and redd disruption, BAC 3‐pass electrofishing	0	0	0	0	0	9.2
Climate.CR‐Redd+BAC‐3p	CR—3‐ redd disruption, BAC 3‐pass electrofishing	30	0	0	0	0	27.6
Climate.CR‐3p+BAC‐3p	CR—3‐pass mechanical removal and BAC 3‐pass electrofishing	30	0	0	0	0	14.4
Climate.CR‐Harvest+BAC‐3p	CR—incentivized harvest (3× current level) and BAC 3‐pass electrofishing	30	0	0	0	0	25.7

Abbreviations: BACL, Bright Angel Creek lower; BACM, Bright Angel Creek middle; BACU, Bright Angel Creek upper; CR, Colorado River.

^a^ Incentivized harvest levels: 1, current harvest $\hat{p}$ = 0.08 on large adults and $\hat{p}$ = 0.03 on small adults; 3, $\hat{p}$ = 0.24 on small adults and $\hat{p}$ = 0.12 on large adults (triple). Mechanical removal levels Colorado River and Glen Canyon with 3‐pass electrofishing: $\hat{p}$_age0_ = 0.27, $\hat{p}$_juveniles_ = 0.17, $\hat{p}$_small adults_ = 0.17, and $\hat{p}$_large adults_ = 0.30 during spawning season. Mechanical removal levels Bright Angel Creek: with 3‐pass electrofishing (current level of removal, during spawning season) $\hat{p}$_age0_ range = 0.30–0.58, $\hat{p}$_juveniles_ = 0.38–0.74, $\hat{p}$_small adults_ = 0.40–0.79, $\hat{p}$_large adults_ = 0.43–0.85 and with 4‐pass electrofishing $\hat{p}$_age0_ range = 0.44–0.78, $\hat{p}$_juveniles_ = 0.54–0.89, $\hat{p}$_small adults_ = 0.56–0.93, $\hat{p}$_large adults_ = 0.60–0.96. See Appendix S4 for details.

For the CR, we simulated suppression involving incentivized harvest by anglers (harvest), redd disruption (RD), and mechanical removal with boat‐mounted electrofishing (MR). We calculated existing levels of harvest (November 2020 to March 2021 [NPS data]) as an approximate proportion of *K* (2020 abundance estimate) and then tripled the proportional harvest for other scenarios (Figure 2). Simulated RD involved a 50% reduction in egg survival prior to the application of the density‐dependent reproduction function (Korman et al., 2011), and MR proportionately removed CR life stages during the spawning season based on triple‐pass electrofishing capture probabilities ($\hat{p}$) Yackulic et al., 2020).

Bright Angel Creek subpopulation suppression included life stage and electrofishing pass‐specific $\hat{p}$ for each subpopulation (MR) (Table 1) estimated from 3‐pass electrofishing (Healy, Moore, et al., 2022). We also included a scenario with eradication of the BACU subpopulation via chemical piscicides and interception of migrants at weir operations (Healy et al., 2020). We assumed complete disconnection of BAC from CR immigration during weir operations.

We simulated a rapid response (RR) to a new brown trout invasion with combinations of likely suppression approaches (MR and RD) applied over 15 years to the CR as a small subpopulation growing toward *K* (Table 1). For RR, we did not apply concurrent treatments to the BAC subpopulations to allow for maximum dispersal to the CR. We compared the population trajectories in the RR scenario with a scenario with similar suppression intensity applied to the stable subpopulation at *K*.

**FIGURE 3:**
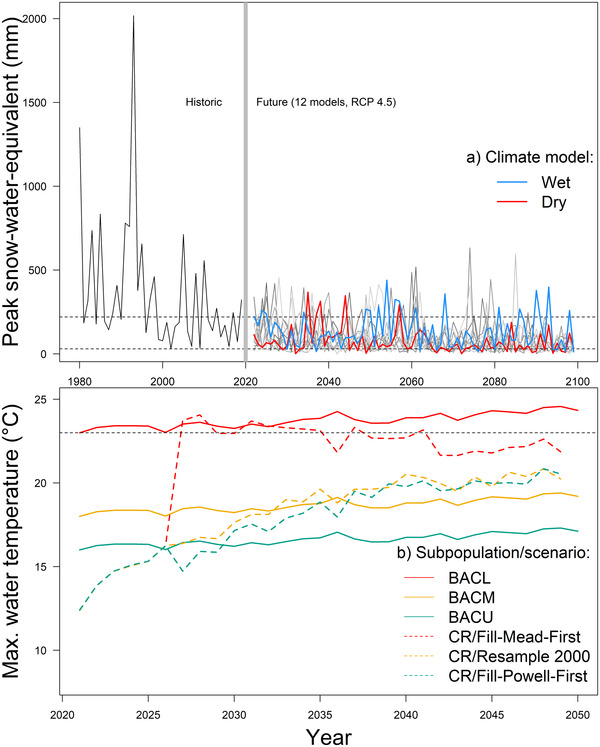
(a) Historic and future peak snow water equivalent (peak SWE) (left and right of the gray vertical bar) from downscaled water balance models (Tercek et al., 2021) (red and blue, selected models used in the individual‐model projection approach; red, high peak spring runoff variability; blue, low peak spring runoff variability; peak SWE values above or below black dashed lines, occurrences of extreme high and low April peak discharge volumes in BAC based on hypothetical relationships between peak SWE and peak spring discharge) and (b) trends in maximum water temperature for Bright Angel Creek (BAC) (methods in Appendix S3) and Colorado River (CR) subpopulations under 3 different simulated climate change scenarios, including fill Mead first (FMF), fill Powell first (FPF), and baseline 2000 resample (data source: Wheeler et al. [2021])

We simulated the effects of 6 hypothetical climate futures on brown trout transition rates by varying hydrology and maximum temperatures (Wenger et al., 2011) for 30 years based on variation in predicted maximum Glen Canyon Dam discharge temperatures (Wheeler et al., 2021) and future BAC spring discharge variability and water temperature (Figure 2). Extreme spring peak discharge volumes limit age 0 brown trout recruitment (Lobón‐Cerviá et al., 2018). Thus, for BAC, we adjusted future spring (February–April) discharge based on projected temperature and precipitation inputs from 2 CMIP5 project models (RCP 4.5 emission scenarios) (Figure 3a) that represented high and low interannual variability in spring snowmelt runoff (hereafter, dry and wet scenarios) (methods in Tercek et al. [2021]). We adjusted maximum annual BAC temperatures based on projected air temperature increases (2.8°C increase) (Tillman et al., 2020) and accounted for longitudinal variation in temperature for each subpopulation (Bair et al., 2019; Appendix S3).

Future temperatures in the CR will vary as a consequence of reservoir water storage decisions and climate change (Udall & Overpeck, 2017; Wheeler et al., 2021). We used projected maximum annual dam discharge temperatures based on recent observed trends (baseline 2000) and potential reservoir storage options prioritizing storage in Lake Powell (upstream, fill Powell first) or Lake Mead (downstream, fill Mead first) reservoirs (Figure 3b) (Wheeler et al., 2021). Finally, we simulated 8 metapopulation suppression scenarios that included combinations of suppression tools applied under the baseline 2000 resample scenario for the CR and wet hydrology for BAC (Table 1; Figure 2).

**FIGURE 4:**
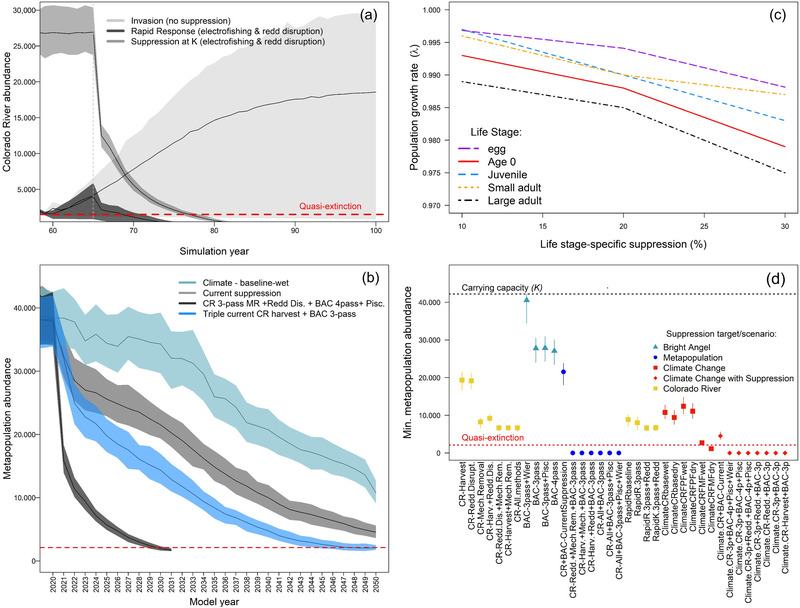
Results of brown trout metapopulation viability simulations for suppression and climate change scenarios, including (a) a comparison of trends in Colorado River (CR) subpopulation abundance during early stages of invasion and following rapid response suppression or suppression of a stable subpopulation at carrying capacity (*K*) (*K* = 5%), (b) relative comparisons of simulated metapopulation abundance under the baseline 2000 climate change scenario with and without suppression (*K* = 5%), (c) perturbation analysis to assess sensitivity of the CR subpopulation to life‐stage‐specific suppression, and (d) minimum metapopulation abundance for all scenarios (*K* = 5%) (red dashed lines, quasi‐extinction threshold; error bands and bars, 5th and 95th percentiles of 100 model runs). Suppression scenario codes in panel (d) and tool combinations are in Table 1.

**FIGURE 5:**
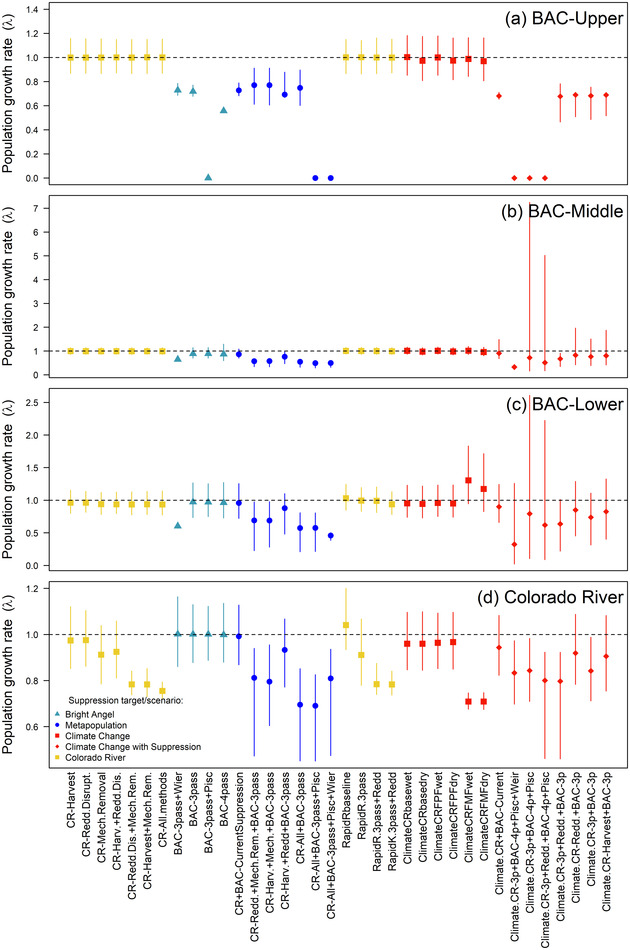
Comparisons of brown trout subpopulation growth rate (*λ*) outcomes for each metapopulation viability model simulation for suppression and climate change scenarios, by subpopulations (a) Bright Angel Creek upper (BAC‐Upper), (b) Bright Angel Creek middle (BAC‐Middle), (c) Bright Angel Creek lower (BAC‐Lower), and (d) Colorado River (error bars, 5th and 95th percentiles of 100 model runs). Scenarios and codes identifying each scenario are described in Table 1.

We compared relative scenario outcomes with subpopulation growth rates (*λ*) during suppression, time to quasi‐extinction (QE, defined as abundance at 5% of *K*), and minimum metapopulation densities (*N*_min_). We also conducted a life‐stage perturbation analysis by simulating 10%, 20%, and 30% suppression of each life stage by itself while holding others constant and comparing median *λ* during suppression (30 years). We focused perturbation analysis on the CR because different techniques may be available to target different life stages (e.g., dam operations to target incubating eggs [Korman et al., 2011] vs. electrofishing for older life stages). All life stages are susceptible to electrofishing in BAC (Healy, Moore, et al., 2022). Although the DyHDER accounts for some environmental and demographic stochasticity (i.e., vital rate temporal variance) (Murphy et al., 2020) (Appendix S1; Figures 4, 5, & 7), we acknowledge our use of mean values for other parameters (e.g., $\hat{p}$ for electrofishing suppression) causes underrepresentation of error in our results; thus, scenario outcomes should be interpreted relative to each other (Morris & Doak, 2002).

## RESULTS

The most effective metapopulation suppression scenarios included combinations of all CR suppression methods with current BAC electrofishing (5.7 or 6.3 years to QE) (Table 1; Figure 4). Of 18 suppression scenarios under existing climatic conditions (excluding rapid response), 6 led to a 100% likelihood of QE—all scenarios applying suppression across the metapopulation led to QE, with the exception of 2020–2021 levels of harvest and 3‐pass BAC electrofishing (Figure 4d; Table 1). Scenarios without MR, the only modeled method targeting age 0 and older life stages in the CR, reduced the probability of QE to 29% and prolonged the time to QE to >27 years (Table 1). Scenarios with MR combined with RD or harvest (triple 2020–2021 levels) led to similar metapopulation suppression (∼10 years to QE). In contrast to the CR, varying suppression intensity applied to BAC subpopulations led to similar metapopulation‐scale outcomes, with the exception of a scenario severing BAC subpopulations from CR immigrants through the use of a weir that delayed metapopulation QE (Table 1). Nonetheless, the weir reduced *λ* for the BACL and BACM subpopulations (Figure 5b,c), demonstrating the importance of connectivity and dispersal to maintaining the BAC subpopulations and overall metapopulation resilience. Maintenance of *N*_min_ near *K* also provided evidence that the weir caused additional dispersal to the CR (Figure 4d) because it did not trap and remove fish in our model. With a waterfall barrier preventing immigration into BACU from downstream subpopulations, we found BACU *λ* < 1 for all BAC suppression scenarios despite lower effectiveness of electrofishing there relative to BACM and BACL (Figure 5).

Based on perturbation analyses, we predicted the CR subpopulation to be most sensitive to large adult and age 0 life stage suppression, which reduced mean *λ* to 0.975 and 0.979, respectively, from a stable *λ* (*λ* = 1), when 30% suppression was applied (Figure 4c). Of the 3 suppression tools individually applied to the CR over 30 years, RD (∼reduced egg survival) and harvest by anglers—both actions targeting a limited number of life stages—were the least effective in reducing *λ* and metapopulation abundance (Table 1; Figures 4d & 5).

Suppression applied to a newly invading CR subpopulation was predicted to reduce the time to QE (median 4 years, 5th and 95th percentiles, 1–7 years) compared with suppression starting with density at *K* (median 12 years to QE, 5th and 95th percentiles, 11–13 years) (Figure 4a,b). Ultimately, targeting multiple life stages with a combination of approaches was important to quickly eliminate the subpopulation. A rapid response with only MR had a minimal effect on λ (0.91, 0.78–1.07), relative to the scenario with MR and 50% RD added (*λ* declined to 0.78, 0.74–0.84) (Figure 5d).

**FIGURE 6:**
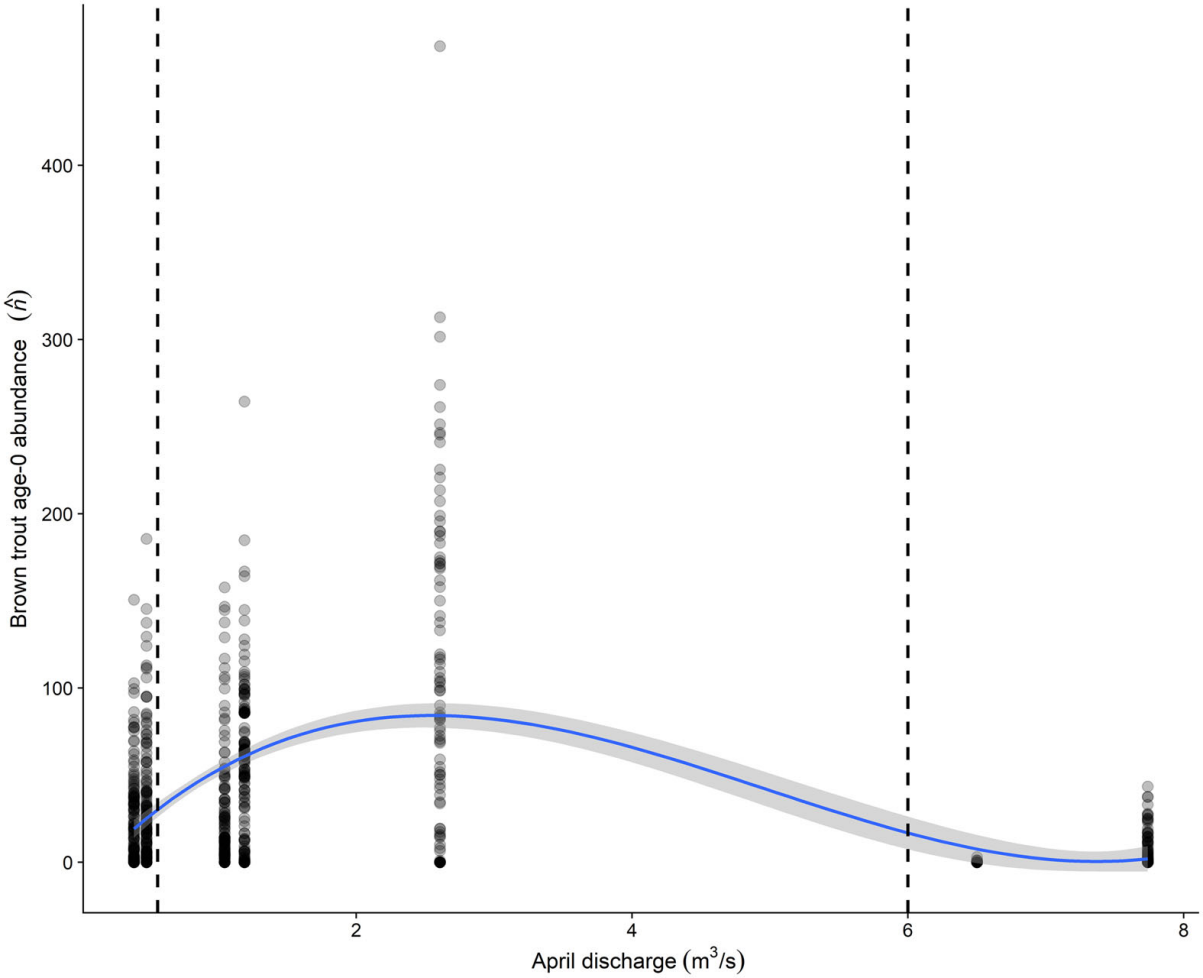
Results of linear‐mixed effects modeling, showing the relationship between April Bright Angel Creek peak discharge magnitude and age 0 brown trout abundance estimated the following fall (points, station‐specific abundance estimates; dashed vertical lines, thresholds for extreme low [left] and high [right] discharge modeled in climate change scenarios)

We found similar support for 3 linear mixed‐effects models representing relationships between peak spring discharge and age 0 brown trout abundance (ΔAICc < 0.5) and no support for models with covariates representing summer temperature, rainbow trout abundance, or winter or monsoon discharge (ΔAIC_c_ > 6) (Appendix S3). The best model among those tested (*R* ^2^ = 0.30) included a third‐order polynomial of April maximum discharge (Figure 6). Age 0 abundance was reduced following years with relatively high or low April discharge—this nonlinear flow–recruitment relationship was included in the mPVA as an age 0 survival optimality curve and, along with maximum summer temperature, formed the basis of our future climate change scenarios for BAC discussed above.

**FIGURE 7:**
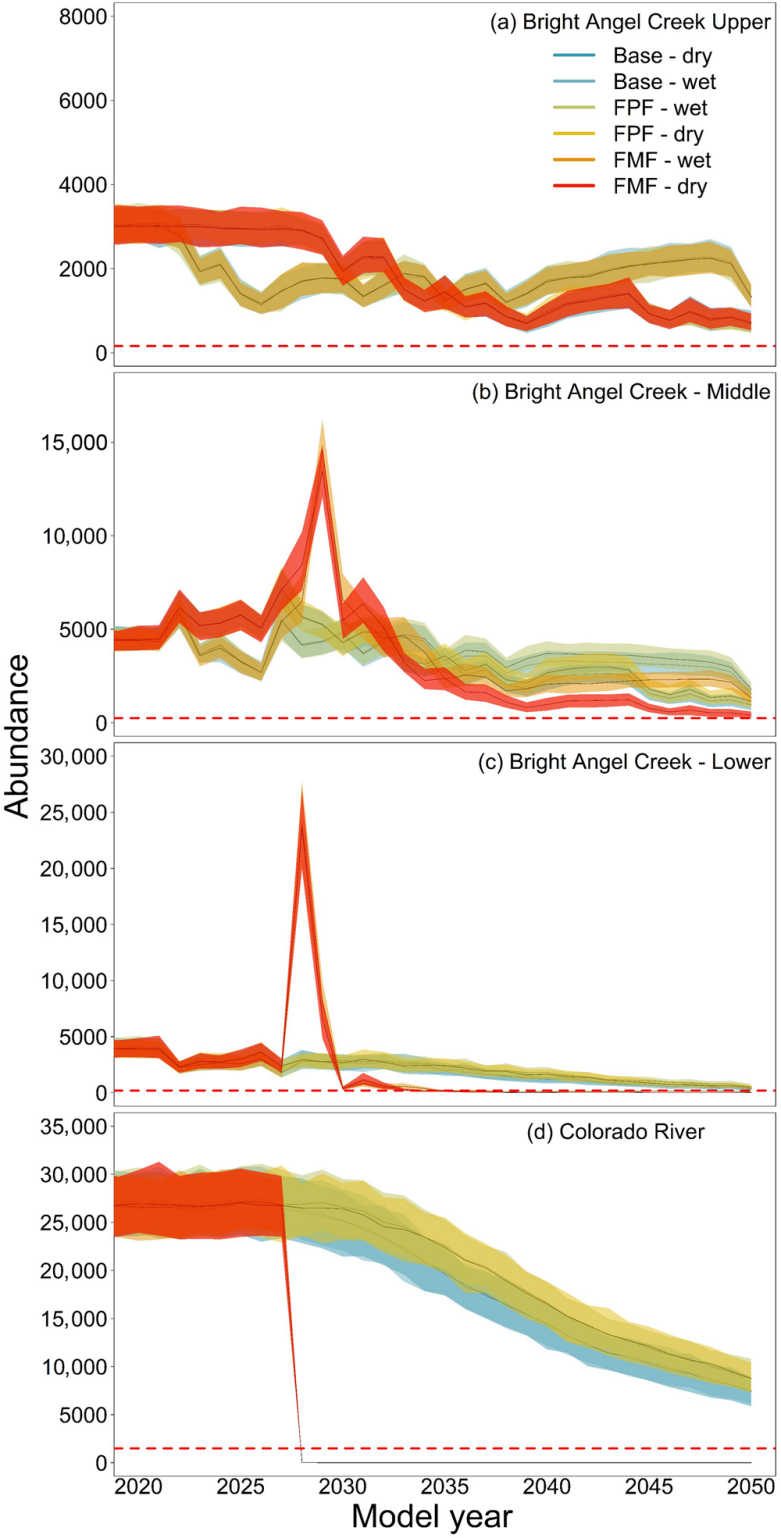
Time series of subpopulation abundance of brown trout in (a–c) Bright Angel Creek and the Colorado River (d) under 6 future climate change scenarios

All future climate scenarios led to eventual declines in the metapopulation, although metapopulation QE was reached for only the fill‐Mead‐first‐dry BAC scenario (16.3 years to QE; Figures 4 & 7; Table 1). The metapopulation *N*_min_ for climate scenarios ranged from 3% to 29% of *K* with the largest reduction in fill Mead first scenarios, followed by the baseline2000 dry scenario (Table 1). The BACU and BACM subpopulations remained above QE for all climate scenarios, whereas CR and BACL subpopulations fell below QE only under fill Mead first (Figure 7). Rapid warming under the fill Mead first reservoir storage scenarios exceeding our assumed thermal limit for brown trout growth (>23°C) led to an abrupt and short‐lived dispersal pulse to BAC, preceding the decline and CR and BACL subpopulation QE. All subpopulations remaining above QE nonetheless declined steadily to the end of the modeled times series (range of *N*_min_ for fill Powell first or baseline2000 scenarios: 8–37% of *K*) (Figure 7; Table 1).

We demonstrated the brown trout metapopulation could persist through 2050 under a plausible future climate change scenario (baseline2000‐wet) with 2020–2021 suppression levels; however, QE was reached for all other scenarios we simulated with higher suppression intensities (Figure 4d). We found MR and RD in the CR applied in combination with BAC stream‐wide 3‐pass electrofishing or 4‐pass electrofishing applied only to BACM and with BACU piscicide application resulted in similar outcomes. Despite these 2 scenarios reaching QE in ∼9 years (Table 1), they represented much different levels of effort. We assumed future temperatures in BACL exceeding 23°C would forego the need for suppression, but with piscicide use additional suppression would be required only in BACM, where future temperatures would remain suitable. Without MR, scenarios with only RD or harvest (at triple the current levels) in the CR and current BAC suppression led to QE in >25 years (Table 1; Figure 4d).

## DISCUSSION

Our metapopulation PVA demonstrated important opportunities and limitations of brown trout suppression approaches. We found QE could be achieved through a sustained metapopulation‐wide suppression program targeting multiple life stages. Conversely, scenarios that did not affect the age 0 life stage across the metapopulation were least effective. Forgoing suppression at 1 subpopulation could also negate the effects of suppression of another through dispersal of individuals from the unsuppressed subpopulation. We also demonstrated advantages of a rapid response to stem invasions, as other authors have (Simberloff, 2003; van Poorten et al., 2019). Lastly, by exploring metapopulation dynamics related to hypothetical habitat changes arising due to climate change and reservoir storage decisions, we observed that declines in the metapopulation were primarily driven by warming CR water temperatures. Some future scenarios may lessen the need for CR suppression, but BAC subpopulations would persist.

Resilience of a metapopulation may depend on local environmental variation, synchrony of dynamics in distinct subpopulations, and connectivity between them allowing for recolonization following catastrophic events (Elkin & Possingham, 2008; Fausch et al., 2009; Rieman & Dunham, 2000). We demonstrated that understanding metapopulation dynamics and the degree of connectivity can assist with prioritization of invasive species subpopulations for control and provide insights into vulnerabilities that could be exploited to improve the likelihood of suppression (Hock et al., 2016; Pepin et al., 2020). We found relative differences in λ and QE probability across subpopulations depended on connectivity. Without immigrants, the upper tributary subpopulation was more likely to reach QE despite reduced electrofishing effectiveness, relative to electrofishing other BAC subpopulations (Healy, Moore, et al., 2022). Maintenance of the BACL subpopulation occupying marginal habitat was more dependent on immigration; as such, the restriction of CR immigrants using a weir caused a significant subpopulation decline. Left unsuppressed and without weir operation, dispersal from the much larger CR subpopulation could negate BAC suppression efforts, particularly in the lower reaches, which provide important habitat for native fishes (Healy et al., 2020). Our predictions are consistent with others showing the importance of headwater tributary and mainstem connectivity to salmonid persistence (Day et al., 2018; Fausch et al., 2009; Tsuboi et al., 2022), including for brown trout populations, where a single barrier could influence density and population structure in distant tributaries (González‐Ferreras et al., 2019).

Large adults, which we hypothesized would move greater distances than other life stages, also have greater capacity to contribute to reproduction (e.g., Goodwin et al., 2016). Accordingly, our results reflected the importance of controlling highly fecund large adults and age 0 life stages in order to reduce relative *λ*. Destruction of nests with eggs or juveniles was more effective in suppressing invasive smallmouth bass (*Micropterus dolomieu*), relative to angling removal of older life stages (van Poorten et al., 2019). Similarly, our results suggest inclusion of early life stage suppression may be an effective technique to control invasive fishes, especially when age 0 survival is high and an important contributor to population growth (Simard et al., 2020). Brown trout metapopulation sensitivity to age 0 or large adult mortality is unsurprising because fecundity scales allometrically with body size, individual body size is generally correlated with reproductive fitness (including in brown trout [Goodwin et al., 2016]), and salmonid population dynamics are often driven by young‐of‐year survival (e.g., Lobón‐Cerviá et al., 2018; Milner et al., 2003).

We demonstrated a dramatically shorter time frame to QE when suppression was applied early in the invasion process and to multiple life stages and found a plausible range in density‐dependent *λ* and the potential for environmental or demographic stochastic QE (Liebhold & Bascompte, 2003). A rapid response to a newly establishing subpopulation could reduce the risk of ecological damage and lead to more efficient and less costly suppression (Pyšek et al., 2020; Simberloff, 2003; van Poorten et al., 2019), relative to delaying action until after an invader has grown in abundance and dispersed (Bair et al., 2018). Successful invaders often possess life‐history traits that facilitate invasiveness or have well‐studied invasion histories, including brown trout (Kulhanek et al., 2017; Spear et al., 2021), and as we demonstrated, population growth is likely. Salmonids generally demonstrate high potential population growth that provides resilience to catastrophic events through density‐dependent demographic rates or immigration from neighboring populations (Day et al., 2018; Saunders et al., 2015). From a metapopulation perspective, the extent of high‐quality salmonid habitat in the Glen Canyon Dam tailwater has the potential to support a much larger subpopulation (potential *K*: 20,000–150,000 [Runge et al., 2018]) that could confer additional metapopulation resilience (i.e., a large “patch” [Hanski, 1998]).

Outcomes of climate change scenarios demonstrated the importance of dispersal and availability of refuge habitats to future metapopulation viability (Elkin & Possingham, 2008; Hanski, 1998). We modeled how changes in the frequency of years with high spring stream discharge or drought may displace fry or reduce habitat space and increase competitive interactions, thereby limiting brown trout recruitment (Cattanéo et al., 2002; Lobón‐Cerviá et al., 2018; this study). Although limited data are currently available to assess drivers of CR recruitment (e.g., effects of current and future flows are unclear), we assumed warming would consistently, negatively affect metapopulation‐wide demographics (Jonsson, & Jonsson, 2009). Subpopulation responses to future scenarios differed due to longitudinal variation in BAC temperatures (Bair et al., 2019), the degree of subpopulation connectivity, and important differences in temperature resulting from reservoir operational decisions. Dispersal between patches is not often considered in metapopulation models applied to invasive species in dendritic stream networks or open systems (Day et al., 2018; van Poorten et al., 2019), despite the importance of dispersal to persistence and vulnerability of stream organisms to fragmentation (González‐Ferreras et al., 2019; Murphy et al., 2020; Tsuboi et al., 2022). Asynchrony in subpopulation dynamics, as we demonstrated for brown trout, could lead to a higher likelihood of long‐term metapopulation persistence (Elkin & Possingham, 2008; Hanski, 1998).

An important finding of our modeling was how declining upstream reservoir storage was predicted to result in dramatic declines in brown trout and the potential loss of the CR subpopulation. The rate of decline in storage may depend on shifts to reservoir water storage prioritization combined with upper basin consumptive water use (Dibble et al., 2021; Wheeler et al., 2021). Similar to our results (high initial dispersal rates to BAC refuges as CR habitat quality declined), others have observed higher rates of dispersal toward refuge patches leading to greater metapopulation viability (Elkin & Possingham, 2008; Tsuboi et al., 2022). Nevertheless, with BAC as a refuge, combined with seasonal diversity in temperature variation provided across the CR‐BAC network (cf. Armstrong et al., 2021; Hahlbeck et al., 2022), the metapopulation could be maintained under even the most severe futures we simulated.

Our conservative approach to modeling metapopulation persistence under future climate change may underestimate the likelihood of brown trout extirpation. For example, we did not include catastrophic events in our simulations that can extirpate tributary fishes in our study region (Healy, Budy, et al., 2022), used an optimistic carbon emission future (RCP 4.5), and assumed BAC baseflows would be maintained. Higher emission scenarios could lead to higher temperatures, more extreme drought, and greater CR flow declines (up to −55%; Udall & Overpeck, 2017). Baseflow declines in BAC due to increased aridity and air temperatures (Tillman et al., 2020) could exacerbate stream warming during summer or fall (Bair et al., 2019), and increased winter rain and flooding could negatively affect spawning adults or incubating eggs, thereby reducing reproductive output (Jonsson, & Jonsson, 2009).

We simulated realistic demographic processes, future environmental stochasticity, and potential management scenarios using a well‐established and parametrized model (DyHDER; Murphy et al., 2020). Nonetheless, we recognize greater uncertainties exist in modeled outcomes than are represented in our results, which are driven by our parameterization choices and information gaps. Our simulations could underrepresent compensatory, density‐dependent survival, and high *λ* under optimum reproductive conditions, which could offset suppression effects (Day et al., 2018; Saunders et al., 2015). Age 0 brown trout increased dramatically in BACU in 2020 despite very low spawning adult densities in 2019 for instance (Appendix S4). Immigration may also increase when stimulated by high flow experiments (Healy, Yackulic, et al., 2022; Runge et al., 2018), which could reduce the effectiveness of a rapid response. Nonetheless, observed declines in BAC subpopulations were generally matched by simulations (Appendix S4; Healy et al., 2020). Although outcomes of scenarios should be viewed relative to each other, rather than as absolutes when considering management options (Morris & Doak, 2002), from a heuristic standpoint, our results should prove useful and support suppression decision‐making.

The 2020–2021 metapopulation‐scale suppression, while effective in temporarily reducing tributary brown trout abundance (Healy et al., 2020), is unlikely to lead to substantial metapopulation‐wide declines, even under plausible climate change scenarios resulting in degraded habitat quality in some subpopulations. Rather, if the goal is to remove invasive brown trout, consistent with management policies (US DOI, 2006), both dramatic increases in angler harvest and additional life stage suppression would need to occur (Dux et al., 2019). Alternatively, managers may face increasingly costly suppression operations to limit dispersal of brown trout to critical endangered fish habitat, where suppression may be less effective (Bair et al., 2018). Uncertainties in participation by anglers, the invulnerability of age 0 fish to angling, and potential economic benefits provided by brown trout (Beever et al., 2019; Nuñez et al., 2012) may hinder angler harvest‐based suppression efforts. Understanding and quantifying operational uncertainty (method effectiveness uncertainty) in suppression techniques, along with biological uncertainties, could improve management outcomes (Li et al., 2021). In addition to angler harvest, operational uncertainties in our study relate primarily to RD or other untested age 0 suppression techniques. Research and development targeting age 0 invasive salmonids, which are generally less vulnerable to common fishing gears, could assist managers in refining suppression programs. For example, we hypothesize that the removal of invasive aquatic vegetation that may provide rearing habitat for age 0 salmonids (Marsh et al., 2021) may limit early life stage survival. Nonetheless, our results suggest expanded CR subpopulation suppression, eradication of isolated climate‐refuge subpopulations, refocusing suppression efforts to other areas with future habitat (e.g., BACM), and ensuring isolation of BAC from CR could limit metapopulation persistence.

Given the worldwide prevalence of ecological damaging salmonids and other invasive fishes (Hansen et al., 2019; McIntosh et al., 2011; Toussaint et al., 2016), our results are broadly applicable to aquatic ecosystem conservation. Our metapopulation PVA approach is novel in that it allowed for the simulation of variation in dispersal and connectivity while accounting for realistic spatial and temporal heterogeneity in physical habitat (Murphy et al., 2020), in the context of invasive species management. The DyHDER model could easily be applied to a more complex interconnected system in which invasive species eradication is perceived as difficult or impossible or include other subpopulations that could result from brown trout expansion in our system. Functional eradication (the suppression level effectively maintaining highly valued ecological services or species) may be feasible even in difficult situations (Green & Grosholz, 2021). For instance, only ∼60% trout reduction may be necessary to maintain BAC native fish populations (Healy et al., 2020).

We also demonstrated how predicting invasive species’ distributional range constrictions or expansions (of warmwater species) with climate change could assist in prioritization of subpopulations for monitoring or response planning (Rahel & Olden, 2008). Relatively large, high‐quality habitat patches would support rapid subpopulation establishment and future metapopulation resilience in the Grand Canyon—patches with similar attributes could be prioritized for early detection monitoring or targeted suppression (Simberloff, 2014). Suppression of established invasive species may not be universally appropriate; however, we suggest that national parks and protected areas, where invasive species control and conservation of endemic species and naturally functioning ecosystems are legally mandated (US DOI, 2006; reviewed in Reaser et al., 2020), be considered top priority for active prevention, rapid response, and suppression of invasions (Buktenica et al., 2013; Lawrence et al., 2011; reviewed in Beever et al., 2019). Our spatially explicit metapopulation approach can assist managers and conservationists in strategically prioritizing costly and often logistically challenging invasive species suppression, particularly in open systems (Hock et al., 2016; Pepin et al., 2020; van Poorten et al., 2019).

## Supporting information

Appendix S1: Model description
Appendix S2: Model parameterization
Figure S1. Relationship between female brown trout fork length and fecundity
Table S1. Subpopulation‐specific demographic parameters and source of information included in the brown trout metapopulation viability model
Appendix S3: Linear mixed‐effects modeling results for brown trout recruitment and simulation scenario development
Table S2. Rankings of linear mixed‐effects models representing hypothesized relationships between environmental drivers of age‐0 brown trout abundance (BNTyoyNhat) in Bright Angel Creek
Table S3. Description of suppression scenarios, intensity levels of suppression actions, and minimum metapopulation or subpopulation abundance (N_min_) over 30 years, and time to quasi‐extinction (QE) for each scenario
Figure S2. Relationship between peak snow‐water‐equivalent, generated using a water balance model (Tercek et al. 2022), and peak spring Bright Angel Creek discharge (USGS gage 09403000 data)
Figure S3. Monthly max air temperatures using Tillman et al. (2020) data from USGS website (converted to average from 370 grids, then to Celsius)
Table S4. Water temperature modeling results using maximum air temperature from Tillman et al. (2020)(MaxMnAnnTemp), and proportional increases (inc) applied to Phantom Ranch baseline air temperature (PRair; 35°C), which were used to generate subpopulation‐specific proportional increases in water temperatures (right 3 columns) using the Bair et al. (2019) water temperature model from baselines of 16°C, 18°C, and 23°C for BACU, BACM, and BACL, respectively
Table. S5. Results of climate change scenario sensitivity analysis, involving adjustment of peak snow‐water‐equivalent and peak spring Bright Angel Creek discharge relationships so that dry and wet scenario thresholds are +/−10% or +/‐ 20% of the baseline
Appendix S4: Validation of the PVA results using observed vs simulated trends in Bright Angel Ceek abundance
Table S6. Cumulative capture probability ($\hat{p}$) values used for BAC brown trout subpopulation suppression scenarios for 2‐4 electrofishing passes
Figure S4. Validation plots of observed for reaches 1, 3, and 5 through spring 2021 (Healy et al. 2020, NPS unpublished 2020‐21 abundance data) compared to simulated trends in normalized abundance (abundance trends normalized to % of carrying capacity) for BACL, BACM, BACU subpopulations
