# Supplementary material for: Exploring metapopulation‐scale suppression alternatives for a global invader in a river network experiencing climate change
Source: Conserv Biol. 2022 Dec 30;37(1):e13993. doi: 10.1111/cobi.13993 (PMC10107352; doi:10.1111/cobi.13993)
Supplement: Supplementary file 1 — Appendix S1: Model description Appendix S2: Model parameterization Figure S1. Relationship between female brown trout fork length and fecundity Table S1. Subpopulation‐specific demographic parameters and source of information included in the brown trout metapopulation viability model Appendix S3: Linear mixed‐effects modeling results for brown trout recruitment and simulation scenario development Table S2. Rankings of linear mixed‐effects models representing hypothesized relationships between environmental drivers of age‐0 brown trout abundance (BNTyoyNhat) in Bright Angel Creek Table S3. Description of suppression scenarios, intensity levels of suppression actions, and minimum metapopulation or subpopulation abundance (Nmin) over 30 years, and time to quasi‐extinction (QE) for each scenario Figure S2. Relationship between peak snow‐water‐equivalent, generated using a water balance model (Tercek et al. 2022), and peak spring Bright Angel Creek discharge (USGS gage 09403000 data) Figure S3. Monthly max air temperatures using Tillman et al. (2020) data from USGS website (converted to average from 370 grids, then to Celsius) Table S4. Water temperature modeling results using maximum air temperature from Tillman et al. (2020)(MaxMnAnnTemp), and proportional increases (inc) applied to Phantom Ranch baseline air temperature (PRair; 35°C), which were used to generate subpopulation‐specific proportional increases in water temperatures (right 3 columns) using the Bair et al. (2019) water temperature model from baselines of 16°C, 18°C, and 23°C for BACU, BACM, and BACL, respectively Table. S5. Results of climate change scenario sensitivity analysis, involving adjustment of peak snow‐water‐equivalent and peak spring Bright Angel Creek discharge relationships so that dry and wet scenario thresholds are +/−10% or +/‐ 20% of the baseline Appendix S4: Validation of the PVA results using observed vs simulated trends in Bright Angel Ceek abundance Table S6. Cumulative capture probab [file COBI-37-0-s001.docx]

**Exploring metapopulation-scale suppression alternatives for a global invader in a river network experiencing climate change**

**Supplementary Information**

Disclaimer: Any use of trade, firm, or product names is for descriptive purposes only and does not imply endorsement by the U.S. Government.

***Appendix S1: Model description***

The model operates by generating Lefkovitch (i.e., matrix based on ontogenetic stages, *S_i_*) matrices for each subpopulation using stage-specific demographic rates, multiplied by habitat suitability (HS) values (*Ψ_t_*) at each time step (*t*). Reproductive rates (*F_i_*) are adjusted depending on the density (*N*) of each subpopulation at the end of *t* relative to the carrying capacity (*K*), where *F_i_* decrease as *N* approaches *K* (i.e., *S_0_* [*N/K*=1]). Prior to the projection of the matrices into the next (annual) timestep, temporal variance is applied to the demographic rates (standard deviation of reproductive rate *σF_i_*, and survival rates *σ_i_*), and subpopulations that fall below a predetermined quasi-extinction (QE) probability are terminated. For all simulations, we set QE for the metapopulation and subpopulations to 5% of the *K*, or 10 individuals, whichever is greatest (see Murphy et al. 2020 for discussion of Allee effects). The next step in the projection involves simulated dispersal of individuals across subpopulations as a function of time-specific *Ψ_t_*, connectivity, distances between sites, and stage-specific dispersal propensity (*q_i_*). Dispersal probabilities increase as *Ψ_t_* approaches zero and *N* increases in a subpopulation. Harvest or suppression scenarios are applied in the final step of the model following the application of the dispersal function. We included an additional function in the model for invasive species suppression that allows for the removal of a proportion ($\hat{p}$) of a life stage representing stage-specific gear capture efficiency generated from mark-recapture (CR, see methods in Yackulic et al. 2020), or depletion data (Healy et al. 2020, BAC, see methods in Healy et al. 2022), or estimated from angler harvest data for CR (https://www.nps.gov/glca/planyourvisit/brown-trout-harvest.htm).

***Appendix S2: Model parameterization***

Our modeled brown trout metapopulation included 4 sub-populations, including upper (BACU), middle (BACM), and lower (BACL) reaches of BAC, and the CR reach between Glen Canyon Dam and the Paria River (see Fig.1 in main text). Brown trout are relatively rare in other tributaries (Runge et al. 2018) and the mainstem Colorado River in Grand Canyon (Rogowski & Boyer 2019); based on recent electrofishing surveys completed by the National Park Service (NPS unpublished 2020-2021 data) showing relatively low catch rates of brown trout in multiple tributaries (Nankoweap, Clear, Crystal, Pipe, Shinumo, and Tapeats Creeks – those with suitable habitat for brown trout), we assumed BAC and the CR reach of Glen Canyon were primary areas of reproduction. All sites are accessible to brown trout dispersing between subpopulations, except the BACU subpopulation is upstream of a waterfall impassable to movement of fish, and movement to the site was accordingly restricted in the model. The CR site is ~147 km from BACL, and BACM and BACU are 4.3 and 9.5 km from BACL. We considered baseline BAC abundance estimates from 2012 (Healy et al. 2020) as carrying capacity (*K*) for each subpopulation, with the exception of BACL, where we adjusted *K* upward to 4000 to stabilize the model. The CR *K* is less understood since the subpopulation is newly establishing (Runge et al. 2018), and continues to grow. We approximated *K* assuming that the most recent population estimates are near carrying capacity (see main text Table S1). We defined 4 life stages of brown trout based on fish size-at-maturity observed during annual spawning season suppression activities in BAC, beginning in 2010 (Healy et al. 2020). Adult brown trout spawn during fall-winter months and eggs hatch and fry emerge from gravels sometime during spring. We considered age-0 brown trout those <175 mm total length (TL), fish from 176-230 mm as the juvenile life stage, small adults were between 231-300, and large adults were >300 (median sizes by life stage = 133, 200, 275, 420 mm TL).

Where we lacked empirical data to estimate vital rates, we used literature-derived estimates for introduced lotic brown trout population; survival rates of 0.15 for age-0 (*S_0_*), and 0.38-0.42 in older life stages (*S_1-3_*) resulted in a stable baseline (i.e., λ ~1) in modeled BAC subpopulations (Table 1) and were within the range reported in the literature (Budy et al. 2008; Grossman et al. 2017). Due to disturbance by visitors, including angling, and habitat limitations, we expected BACL to have slightly lower age-0 (*S_0_* =0.04), and juvenile and adult survival (*S_1-3_* =0.2). Survival rates for age-1 to large adult CR brown trout were estimated using methods in Yackulic et al. (2020). Age-0 survival in the CR was adjusted (to *S_0_* =0.16; Table S1) to stabilize the CR population near *K*. While *S_0_* =0.16 may appear higher than other published estimates in established populations (e.g., up to 0.08%, Jorgensen & Berg 1991), early life stage survival is often difficult to estimate, can drive populations of invasive species, and may exceed 6 times the rate in an invasive salmonids’ native range (Syslo et al. 2020).

We calculated reproductive rates (*F*) using size-specific fecundity based on egg counts for each reproductive age-class (small adult = 368 eggs/female, *F_3_* = 11; large adults 1699 eggs/female, *F_4_*=51) and a female : male ratio of 0.3 from brown trout sacrificed in BAC, multiplied by an egg-to-fry survival of 0.10 (range 0.053 - 0.10; Syrjänen et al. 2017). Fecundity was assigned to each reproductive age class using egg counts from brown trout sacrificed in BAC by one of the authors (mode for small adults = 275 mm, 368 eggs/female; large adults = 420 mm, 1699 eggs/female), and an egg-fork length relationship (Fig. S1; 0.3 females x 0.1 egg-to-fry survival x 368 or 1699 eggs/female = 11 or 51). It was difficult to decide what mean size to use for large adults since mean sizes range from 280-560 mm between ages 3 and 6+, as estimated using mixed distribution models (Macdonald & Pitcher 1979), and we assumed 420 mm would represent commonly-sized large migratory adults (larger adults were rare).

Brown trout stage-specific dispersal rates and life-history strategies are highly variable and flexible, with both migratory and resident life history expressions common within a single population (Cucherousset et al. 2005; Goodwin et al. 2016; Birnie-Gauvin et al. 2019). Movement probabilities (*q*) were available for small and large adult brown trout (*q_small, large adult_* = 0.016) in our study metapopulation from a 20-year mark-recapture dataset (Healy et al. 2023*.*). Lacking data for early life-stages, we scaled *q_age-0_,_juvenile_* assuming higher dispersal probabilities of age-0 (*q_age-0_* = 0.10; Vatland & Caudron 2015) and juvenile brown trout (*q_juvenile_* = 0.05) than adults (cf. Ciepiela & Walters 2019). We lacked stage-specific dispersal distances; however, we have recaptured or detected large brown trout in BAC that were tagged in the CR (>147 km away), indicating long-distance dispersal is possible. We scaled dispersal distance to 500 m for age-0 fish (Vøllestad et al. 2012; Eisenhauer et al. 2020), and then set distance scalers for juveniles, small adults, and large adults to 4, 12, 20 km assuming larger fish would move longer distances (Radinger & Wolter 2014). We borrowed process variance estimates, transition rates, and parameters to adjust density-dependent reproductive rates (i.e., Beverton-Holt parameters) from Murphy et al. (2020) based on a long time series of empirical data.

*Figure S1. Relationship between female brown trout fork length and fecundity.*

Table S1. Subpopulation-specific demographic parameters and source of information included in the brown trout metapopulation viability model.

| Model parameter | Subpopulation-specific parameters | | |  |
| --- | --- | --- | --- | --- |
| Stage-specific survival*^a^* | Bright Angel Creek lower | Bright Angel Creek middle | Bright Angel Creek Upper | Colorado River |
| Age-0*, S*_0_ | 0.04 | 0.15 | 0.15 | 0.16 |
| Juvenile*, S*_1_ | 0.2 | 0.38 | 0.42 | 0.3 |
| Small adult*, S*_2_ | 0.2 | 0.38 | 0.42 | 0.3 |
| Large adult, *S*_3_ | 0.2 | 0.38 | 0.42 | 0.74 |
| Transition rates*^b^* |  |  |  |  |
| *g*_01_ | 1 | 1 | 1 | 1 |
| *g*_12_ | 0.5 | 0.5 | 0.5 | 0.5 |
| *g*_23_ | 0.5 | 0.5 | 0.5 | 0.5 |
| Reproduction  rates*^c^* |  |  |  |  |
| *F*_0_ | 0 | 0 | 0 | 0 |
| *F*_1_ | 0 | 0 | 0 | 0 |
| *F*_2_ | 11 | 11 | 11 | 11 |
| *F_3_* | 51 | 51 | 51 | 51 |
| Temporal  variance*^b^* |  |  |  |  |
| *s_S_*_0_ | 0.03 | 0.03 | 0.03 | 0.03 |
| *s_S_*_1_ | 0.03 | 0.03 | 0.03 | 0.03 |
| *s_S_*_2_ | 0.03 | 0.03 | 0.03 | 0.03 |
| *s_S_*_3_ | 0.03 | 0.03 | 0.03 | 0.03 |
| *σF*_0_ | 0.00 | 0.00 | 0.00 | 0.00 |
| *σF*_1_ | 0.00 | 0.00 | 0.00 | 0.00 |
| *σF*_2_ | 0.90 | 0.90 | 0.90 | 0.90 |
| *σF*_3_ | 1.50 | 1.50 | 1.50 | 1.50 |
| Carrying  capacity*^d^* (*K*) | 4000 | 5000 | 3200 | 30000 |
| Density-  dependent  parameters*^b^* | |  |  |  |
| *S*_0_ *(N/K*=1*)* | 0.05 | 0.05 | 0.05 | 0.05 |
| *S*_0_ *(N/K*~0*)* | 0.15 | 0.15 | 0.15 | 0.15 |
| Dispersal*^e^* |  |  |  |  |
| *q*_Age-0_ | 0.1 | 0.1 | 0.1 | 0.1 |
| *q*_Juvenile_ | 0.05 | 0.05 | 0.05 | 0.05 |
| *q*_small adult_ | 0.016 | 0.016 | 0.016 | 0.016 |
| *q*_Large adult_ | 0.016 | 0.016 | 0.016 | 0.016 |
| Distance  scalars for  movement  (km)*^f^* | |  |  |  |
| Age-0 | 0.5 | 0.5 | 0.5 | 0.5 |
| Juvenile | 4 | 4 | 4 | 4 |
| Small adult | 12 | 12 | 12 | 12 |
| Large adult | 20 | 20 | 20 | 20 |
|  |  |  |  |  |

^a^Bright Angel Creek (BAC) and age-0 Colorado River (CR) stage-specific survival rates informed by literature as described in Appendix S2 test, and rates set to achieve a stable population abundance baseline. Mark-recapture estimates for CR survival rates (other than age-0) based on methods of Yackulic et al. (2020).

^b^From Murphy et al (2020).

^c^Calculated from size-specific fecundity based on egg counts from sexually mature brown trout removed from BAC and a female:male ratio of 0.3 (National Park Service data). Assumptions: mean size of small adults, 275 mm; large adults, 420 mm; egg-fry survival rate 0.1. *F* = 0.3 female:male ratio x 368 or 1699 eggs/female x 0.1 egg-fry survival rate. See Appendix S2 text.

^d^Baseline abundance from Healy et al. (2020) for Bright Angel Creek middle (BACM) and Bright Angel Creek upper (BACU) subpopulations. The 2020 estimate for CR calculated as in Yackulic et al. (2020). For Bright Angel Creek lower (BACL) carrying capacity was .adjusted upward to achieve stable baseline.

^e^ Healy et al. 2023 average movement probabilities for small and large adults. Literature review for age-0, as described in text in Appendix S2.

^f^Based on best professional judgment and known movements of tagged fish in Grand Canyon. Large fish assumed to move greater distances than small fish.

***Appendix S3: Linear mixed-effects modeling results for brown trout recruitment and simulation scenario development***

*Habitat suitability (optimality) functions–* We incorporated habitat suitability (HS) indices (optimality function, Murphy et al. 2020) into survival and transition rates using both empirical and literature derived mechanistic relationships between brown trout demographic rates and environmental variables. Water temperature (hereafter, temperature) is an important driver of life cycle processes in salmonids, including growth, reproductive rates, and metabolic rates (Railsback & Rose 1999). We used a HS curve (Railsback & Rose 1999) encompassing a liberal range of temperature for growth (5 - 23 °C, Forseth et al. 2009), assuming piscine and macroinvertebrate prey is unlimited in BAC (Whiting et al. 2014), CR brown trout consume high proportions of fish (Yard et al. 2011), and optimum temperatures for growth may be 3-4°C higher for piscivorous trout (reviewed in Jonsson & Jonsson 2009). For instance, optimum temperatures for growth may be 3-4°C higher for trout feeding on fish than those consuming invertebrates (reviewed in Jonsson & Jonsson 2009). To account for observed spatial (BAC, Bair et al. 2019; CR, USGS Gaging Station 09380000) and simulated temporal temperature variation potentially constraining growth, we applied a temperature HS curve to transition rates, with maximum observed summer mean daily temperatures for each subpopulation and scenario. The temperature HS curve applied to brown trout, assuming optimum temperature is 14°C, which is within the range of optimal growth for fry reported in the literature (reviewed in Jonsson & Jonsson, 2009; no growth occurred above 23.1°C; displayed in Figure 6 in Murphy et al. [2020]). Fishes may also distribute themselves to maximize growth based on temperature and resource availability (Hughes & Grand 2000); thus dispersal was also a function of temperature (*Ψ_temp_,_t_*).

*Predictors of age-0 brown trout* – Population dynamics of salmonids are often driven by survival from egg to age-1 (Milner et al. 2003; Lobón-Cerviá 2009). To understand drivers of brown trout recruitment to incorporate into our climate change scenario HS curves, we compared linear mixed effects models including covariates representing hypothesized relationships between environmental variables and abundance of age-0, using data from BAC between 2012-2017 and 2019 (sampling described in Healy et al. 2020). Mixed-effects modeling methods are similar to those described in Healy et al. (2020) to predict native fish abundance in sampling sites, with the exception that zero-inflation model components were excluded and a Poisson error distribution was used for age-0 brown trout counts. Brown trout fry may be sensitive to extreme flow events following absorption of yolk sacs and emergence from among interstitial spaces in spawning gravels (Lobón-Cerviá 2009; Lobón-Cerviá et al. 2018), or to warm summer temperatures (Smialek et al. 2021). We also hypothesized that summer monsoon flooding could lead to reduced fall age-0 brown trout survival and abundance. We tested a model including the coefficient of variation (CV) of maximum daily flow in July-September as a covariate (Healy et al. 2020). Invasive rainbow trout (*Oncorhynchus mykiss*) were the only other large-bodied piscivore present in BAC (Whiting et al. 2014); we included adult (total length >230 mm) rainbow trout abundance as a covariate in our models to test whether large rainbow trout limit brown trout recruitment. Other tested models included covariates representing temperature and seasonal or monthly flow volume (mean monthly or seasonal discharge) and flow variability (CV of monthly or seasonal discharge) during spring and summer emergence (February – May) and growth (June) periods for age-0 fish. We also included the 30-day maximum discharge (Richter et al. 1996) and the number of days >12°C between April and July in separate models. To test for non-linear relationships (Rosenfeld 2017) with age-0 brown trout abundance, we also included 2^nd^ and 3^rd^ order polynomials for models representing discharge hypotheses. Models included on offset term (log electrofishing station length) to standardize variable sampling station lengths (see Healy et al. 2020), and a random intercept for the year corresponding to the sampling year for each station. We used AIC*_c_* to compare models, and considered models within ΔAIC = 2 of the top model to be equally supported (Burnham & Anderson 2002). Results of AIC*_c_* rankings is shown in Table S1. We used the lme4 package (Bates et al. 2015) in R (R Core Team 2019) for linear mixed-effects modeling.

In summary, we applied a temperature HS curve to transition rates in all scenarios and to age-0 survival in climate change scenarios, which also including future temperature variation from climate models. To represent results of linear mixed-effects modeling, we also included April mean discharge (3^rd^ order polynomial) as a second optimality function driving age-0 survival (see Table S2, also see Fig. 3 in the main body of the manuscript). We selected the geometric mean fuzzy aggregation approach in DyHDER when both April discharge and temperature covariates were included in climate change scenarios (Murphy et al. 2020).

| Table S2. Rankings of linear mixed-effects models representing hypothesized relationships between environmental drivers of age-0 brown trout abundance (BNTyoyNhat) in Bright Angel Creek. | | | |
| --- | --- | --- | --- |
| Model | ΔAICc | df | weight |
| BNTyoyNhat ~ April+I(April^2)+I(April^3)+offset(log(station length))+(1\|year) | 0 | 6 | 0.3146 |
| BNTyoyNhat ~ 30day.max+I(X30day.max^2)+I(X30day.max^3)+offset(log(station length))+(1\|year) | 0.1 | 6 | 0.3025 |
| BNTyoyNhat ~ 30day.max+I(X30day.max^2)+offset(log(station length))+(1\|year) | 0.5 | 5 | 0.2505 |
| BNTyoyNhat ~ April+I(April^2)+ offset(log(station length)) +(1\|year) | 3.3 | 5 | 0.06 |
| BNTyoyNhat ~ DaysOver12+offset(log(station length))+(1\|year) | 6.1 | 4 | 0.0151 |
| BNTyoyNhat ~ 1 +offset(log(station length))+(1\| year) | 6.6 | 3 | 0.0113 |
| BNTyoyNhat ~ Adult rainbow troutNhat+offset(log(station length))+(1\|year) | 7.3 | 4 | 0.0081 |
| BNTyoyNhat ~ SpringMnQ+I(SpringMnQ^2)+offset(log(station length))+(1\|year) | 7.3 | 5 | 0.008 |
| BNTyoyNhat ~ DaysOver12+I(DaysOver12^2)+offset(log(station length))+(1\|year) | 7.4 | 5 | 0.0076 |
| BNTyoyNhat ~ February+I(February^2)+offset(log(station length))+(1\|year) | 7.6 | 5 | 0.007 |
| BNTyoyNhat ~ SpringMxCV+I(SpringMxCV^2)+offset(log(station length))+(1\|year) | 7.9 | 5 | 0.0061 |
| BNTyoyNhat ~ June+I(June^2)+offset(log(station length))+(1\|year) | 9 | 5 | 0.0035 |
| BNTyoyNhat~MonsoonMxCV+I(MonsoonMxCV^2)+offset(log(station length))+(1\|year) | 9.3 | 5 | 0.003 |
| BNTyoyNhat ~ March+I(March^2)+offset(log(station length))+(1\|fyear) | 9.5 | 5 | 0.0028 |

*Management scenarios* – We simulated 30-year brown trout suppression scenarios targeting different life-stages across a range of intensity levels including hypothetical and ongoing actions in management plans, a stable baseline (no suppression), and climate change scenarios with and without suppression. We compared outcomes of simulations using subpopulation growth rates (λ) during suppression, and time to quasi-extinction (QE, defined as abundance at 5% of *K*) and minimum metapopulation densities (*N_min_*). We conducted a perturbation analysis to assess life-stage specific sensitivity by simulating 10, 20, and 30% suppression of each life stage by itself while holding others constant, and comparing median λ during suppression (30 years). We focused perturbation analysis on the CR since different techniques may be available to target different life stages (e.g., dam operations to target incubating eggs [Korman et al. 2011], vs electrofishing for older life stages); all life stages are susceptible to electrofishing in BAC [Healy et al. 2022]). To assess the importance of dispersal between subpopulations to metapopulation resiliency, we modeled brown trout suppression in CR and BAC separately while maintaining baseline conditions in the non-suppressed subpopulation, and then applied suppression to all subpopulations concurrently (main manuscript text, Table 1).

We simulated a CR-specific suppression program involving incentivized harvest by anglers (harvest), redd disruption (RD), and mechanical removal using boat-mounted electrofishing (MR) – we included scenarios with actions applied singly and in combination. We simulated existing levels of harvest (November 2020 – March 2021) of vulnerable life stages based on NPS harvest data (https://www.nps.gov/glca/planyourvisit/brown-trout-harvest.htm) as an approximate proportion of the 2020 abundance estimate (capture probability [$\hat{p}],\hat{p}$_small adults_ = 0.03, $\hat{p}$_large adults_ = 0.08), and then tripled the proportional harvest for other scenarios (Table 1). We simulated RD by applying a 50% reduction in egg survival prior to the application of the density-dependance function (Korman et al. 2011), and simulated MR by proportionately removing CR life stages vulnerable to electrofishing by tripling the stage-specific $\hat{p}$ estimates for single-passes of electrofishing throughout the subpopulation ($\hat{p}$ = 1-[1- single pass *p*]^3^ : $\hat{p}$_age-0_ = 0.27, $\hat{p}$_juveniles_ = 0.17, $\hat{p}$_small adults_ = 0.17, $\hat{p}$_large adults_ = 0.30 during spawning season; Yackulic et al. 2020).

Our simulations of BAC subpopulation suppression included life stage- and electrofishing pass-specific $\hat{p}$ for each subpopulation (MR; Table S3) estimated from 3-pass electrofishing (Healy et al. 2022) and validated against observed trout declines (Healy et al. 2020; Appendix S4). We adjusted electrofishing $\hat{p}$ upward to simulate a 4^th^ pass, and included a scenario with simulated eradication of the BACU subpopulation using chemical piscicides (Table S3). To simulate the interception of migratory trout achieved through weir operations (Healy et al. 2020), and to assess the effect of dispersal in maintaining the metapopulation, we assumed complete disconnection of BAC from CR immigration, although this unlikely to be completely true.

We simulated a rapid response (RR) to a new brown trout invasion, where likely suppression approaches (MR and RD) are applied to the CR as a small subpopulation growing toward *K* (Table S3). For these simulations, we set the initial brown trout density to QE, and then applied 3-pass MR alone, and with 50% RD for 15 years once the mean simulated density had grown for 5 years. We did not apply concurrent treatments to the BAC subpopulations to allow for maximum dispersal to the CR. The 15-year RR scenario allowed us to compare the amount of time required and probability of achieving QE with similar suppression intensity applied to the stable subpopulation at *K*.

Table S3. Description of suppression scenarios, intensity levels of suppression actions, and minimum metapopulation or subpopulation abundance (*N_min_*) over 30 years, and time to quasi-extinction (QE) for each scenario.

| Code | Scenario Description | Incent. harvest*^a^* | Redd disrupt. (age-0) | Mech. removal (CR)*^b^* | Mech. removal (BAC)*^c^* | Duration (years) | Meta- population *N_min_* | BACL *N_min_* | BACM *N_min_* | BACU *N_min_* | CR *N_min_* | QE time |
| --- | --- | --- | --- | --- | --- | --- | --- | --- | --- | --- | --- | --- |
|  | Baseline/stable subpopulations at carrying capacity (*K*) | - | - | - | - | - | 37529 | 3811 | 4305 | 2971 | 26317 | - |
| *Colorado River suppression scenarios* | | | | | | | | | | | |  |
| CR-Harvest | Incentivized angler harvest | 3 | - | - | - | 30 | 19334 | 1117 | 3410 | 2998 | 11772 | - |
| CR-Redd.Disrupt | Redd disruption | - | 50% | - | - | 30 | 19162 | 1127 | 3398 | 2995 | 11514 | - |
| CR-Mech.Removal | Mechanical removal – boat-based electrofishing | - | - | 3 | - | 30 | 8240 | 482 | 3201 | 2977 | 1483 | - |
| CR-Harv.+Redd.Dis | Incentivized harvest + redd disruption | 3 | 50% | - | - | 30 | 9180 | 494 | 3214 | 2989 | 2317 | - |
| CR-Redd.Dis.+Mech.Rem | Redd disruption + mechanical removal | - | 50% | 3 | - | 30 | 6679 | 454 | 3172 | 2974 | 12 | - |
| CR-Harvest+Mech.Rem. | Incentivized harvest + mechanical removal | 3 | - | 3 | - | 30 | 6669 | 454 | 3169 | 2990 | 12 | - |
| CR-All.methods | Incentivized harvest + redd disruption+ mechanical removal | 3 | 50% | 3 | - | 30 | 6647 | 459 | 3193 | 2984 | 4 | - |
| *Bright Angel Creek suppression scenarios* | | | | | | | | | | | |  |
| BAC-3pass+Weir | Mechanical removal (3-pass electrofishing) with weir installation | - | - | - | 3 | 30 | 40548 | 0 | 0 | 1 | 30000 | - |
| BAC-3pass | Mechanical removal (3-pass electrofishing) | - | - | - | 3 | 30 | 27795 | 1108 | 50 | 1 | 26617 | - |
| BAC-3pass+Pisc | Mechanical removal (3-pass electrofishing) and piscicide applied to BACU | - | - | - | 3 | 30 | 27844 | 1112 | 49 | 0 | 26647 | - |
| BAC-4passes | Mechanical removal (4-pass electrofishing) stream-wide | - | - | - | 4 | 30 | 27078 | 560 | 8 | 0 | 26510 | - |
| *Metapopulation-scale suppression scenarios* | | | | | | | | | | | | |
| CR+BAC-CurrentSuppression | Incentivized harvest at current level and BAC 3-pass electrofishing stream-wide | 1 | - | - | 3 | 30 | 21527 | 671 | 23 | 0 | 20830 | - |
| CR-Redd.+Mech.Rem.+BAC-3pass | CR - Redd disruption and mechanical removal with 3-pass electrofishing stream wide |  | 50% | 3 | 3 | 30 | 0 | 0 | 0 | 0 | 0 | 10.0 |
| CR-Harv.+Mech.+BAC-3pass | CR-incentivized harvest and mechanical removal with 3-pass stream wide electrofishing | 3 | - | 3 | 3 | 30 | 0 | 0 | 0 | 0 | 0 | 10.3 |
| CR-Harv.+Redd+BAC-3pass | CR-incentivized harvest and redd disruption with 3-pass stream wide electrofishing | 3 | 50% | - | 3 | 30 | 0 | 0 | 0 | 0 | 0 | 27.3 |
| CR-All+BAC-3pass | CR-all suppression methods with 3-pass stream wide electrofishing | 3 | 50% | 3 | 3 | 30 | 0 | 0 | 0 | 0 | 0 | 6.3 |
| CR-All+BAC-3pass+Pisc | CR-all suppression methods with 3-pass electrofishing with piscicides applied to BACU | 3 | 50% | 3 | 3 | 30 | 0 | 0 | 0 | 0 | 0 | 5.7 |
| CR-All+BAC-3pass+Pisc+Weir | CR-all suppression methods with 3-pass electrofishing with piscicides applied to BACU and weir installation | 3 | 50% | 3 | 3 | 30 | 0 | 0 | 0 | 0 | 0 | 11.4 |
| *Rapid response scenarios* | | | | | | | | | | | | |
| RapidRbaseline | CR- baseline unsuppressed growth for comparison to rapid response suppression | - | - | - | - | 40 | 8871 | 549 | 3247 | 2974 | 1608 | - |
| RapidR.3-pass | CR-Rapid response to small/increasing population using boat electrofishing | - | - | 3 | - | 15 | 8043 | 478 | 3155 | 2991 | 1303 | 13 |
| RapidR.3-pass+Redd | CR-Rapid response to small/increasing population using boat electrofishing and redd disruption | - | 50% | 3 | - | 15 | 6645 | 445 | 3134 | 2988 | 65 | 4 |
| RapidK.3-pass+Redd | CR- boat electrofishing and redd disruption applied to stable subpopulation at carrying capacity | - | 50% | 3 | - | 15 | 6708 | 463 | 3198 | 2996 | 12 | 12 |
| *Climate change scenarios* | | | | | | | | | | | |  |
| ClimateCRbasewet | Baseline2000 resample with annual max (based on means of 100 traces), BAC RCP 4.5 SWE and Tillman temp increases, with wetter model | - | - | - | - | 30 | 10771 | 374 | 1575 | 1180 | 7505 | - |
| ClimateCRbasedry | Baseline2000 resample with annual max (based on means of 100 traces), BAC RCP 4.5 SWE and Tillman temp increases, with drier model | - | - | - | - | 30 | 9422 | 335 | 959 | 683 | 7416 | - |
| ClimateCRFPFwet | Baseline2000 resample with FillPowellFirst, BAC RCP 4.5 SWE and Tillman temp increases, with wetter model | - | - | - | - | 30 | 12408 | 528 | 1785 | 1173 | 8796 | - |
| ClimateCRFPFdry | Baseline2000 resample with FillPowellFirst, BAC RCP 4.5 SWE and Tillman temp increases, with drier model | - | - | - | - | 30 | 11100 | 491 | 1152 | 682 | 8775 | - |
| ClimateCRFMFwet | Baseline2000 resample with FillMeadFirst, BAC RCP 4.5 SWE and Tillman temp increases, with wetter model | - | - | - | - | 30 | 2708 | 48 | 1336 | 1141 | 0 | - |
| ClimateCRFMFdry | Baseline2000 resample with FillMeadFirst, BAC RCP 4.5 SWE and Tillman temp increases, with drier model | - | - | - | - | 30 | 1133 | 5 | 407 | 702 | 0 | 16.3 |
| *Suppression scenarios with climate change* | | | | | | | | | | | | |
| Climate.CR+BAC-Current | Current level of suppression with basewet climate scenario | 1 | - | - | 3 | 30 | 4550 | 64 | 45 | 0 | 4439 | - |
| Climate.CR-3p+BAC-4p+Pisc+Weir | CR-3-pass mechanical removal, BACM 4-pass electrofishing, BACU piscicide application and weir installation | - | - | 3 | 4 | 30 | 0 | 0 | 0 | 0 | 0 | 15.7 |
| Climate.CR-3p+BAC-4p+Pisc | CR-3-pass mechanical removal, BACM 4-pass electrofishing, BACU piscicide application | - | - | 3 | 4 | 30 | 0 | 0 | 0 | 0 | 0 | 14.5 |
| Climate.CR-3p+Redd+BAC-4p+Pisc | CR-3-pass mechanical removal and redd disruption, BACM 4-pass electrofishing, BACU piscicide application | - | 50% | 3 | 4 | 30 | 0 | 0 | 0 | 0 | 0 | 9.0 |
| Climate.CR-3p+Redd+BAC-3p | CR-3-pass mechanical removal and redd disruption, BAC 3-pass electrofishing | - | 50% | 3 | 3 | 30 | 0 | 0 | 0 | 0 | 0 | 9.2 |
| Climate.CR-Redd+BAC-3p | CR-3- redd disruption, BAC 3-pass electrofishing | - | 50% | - | 3 | - | 30 | 0 | 0 | 0 | 0 | 27.6 |
| Climate.CR-3p+BAC-3p | CR-3-pass mechanical removal and BAC 3-pass electrofishing | - | - | 3 | 3 | - | 30 | 0 | 0 | 0 | 0 | 14.4 |
| Climate.CR-Harvest+BAC-3p | CR-Incentivized harvest (3X current level) and BAC 3-pass electrofishing | 3 | - | - | 3 | - | 30 | 0 | 0 | 0 | 0 | 25.7 |

*Climate change scenario development and sensitivity analysis –* Historic and future projected future Bright Angel Creek snow-water-equivalent (SWE) estimates were provided by the NPS Climate Change Response Program (NPSCCRP, <https://www.nps.gov/orgs/ccrp/index.htm>) using downscaled climate change projections for a 4 km^2^ grid (1 km^2^ resolution) located on the North Kaibab Plateau. The area selected was assumed to be representative of the groundwater recharge catchment for Roaring Springs (a major source of Bright Angel Creek, Bair et al. 2019). Accumulated daily SWE forecasts for the selected grid from 2022-2099 were estimated by NPSCCRP staff using a water balance model (Tercek et al. 2021) with inputs of future precipitation and temperatures from 12 CMIP5 general circulation models using relative concentration pathway 4.5 (RCP 4.5). We calculated peak SWE from February to May using 2 (INMCM.4, Volodin et al. 2010; BNU.ESM, Ji et al. 2014) of the 12 available models, which were chosen to represent a range of future interannual flow variability (high and low peak flow years). We used 2 selected models to ensure a plausible range of interannual variability was captured, which is lost when model averages are used. This individual (model) projection approach was found to better represent a range in variability in future conditions than a model ensemble approach, for instance (Lawrence et al. 2021). Ideally, we would use relationships between historic peak SWE and peak spring stream discharge to develop high and low flow thresholds driving age-0 brown trout survival; however, past SWE and peak spring discharge (USGS gage 09403000) relationships in BAC (2006-2019) were noisy (e.g., high SWE did not always correlate with high runoff, R^2^= 0.42 see Fig. S2). Spring runoff magnitude in Bright Angel Creek would be driven by many variables and the relationship between the 2 variables are likely complicated by interannual variation in winter rain frequency and amount, air temperature, soil moisture, and others (Stewart 2009; Hammond et al. 2018). We assumed peak SWE >220 and <30 mm would represent high and low flows (~5.7 m^3^/s or 0.57 m^3^/s during spring runoff; see HS curve in Figure 3 in main text), which we represented according to the future projected frequency of high and low SWE. The frequency of high and low spring discharge occurrences was then reflected in time series in climate scenarios based on peak SWE from the 2 chosen models, with all other years’ peak SWE falling between these thresholds assumed to be optimum discharge for age-0 brown trout survival. Results of the sensitivity analysis of these high and low thresholds are described below (and see Table S4).


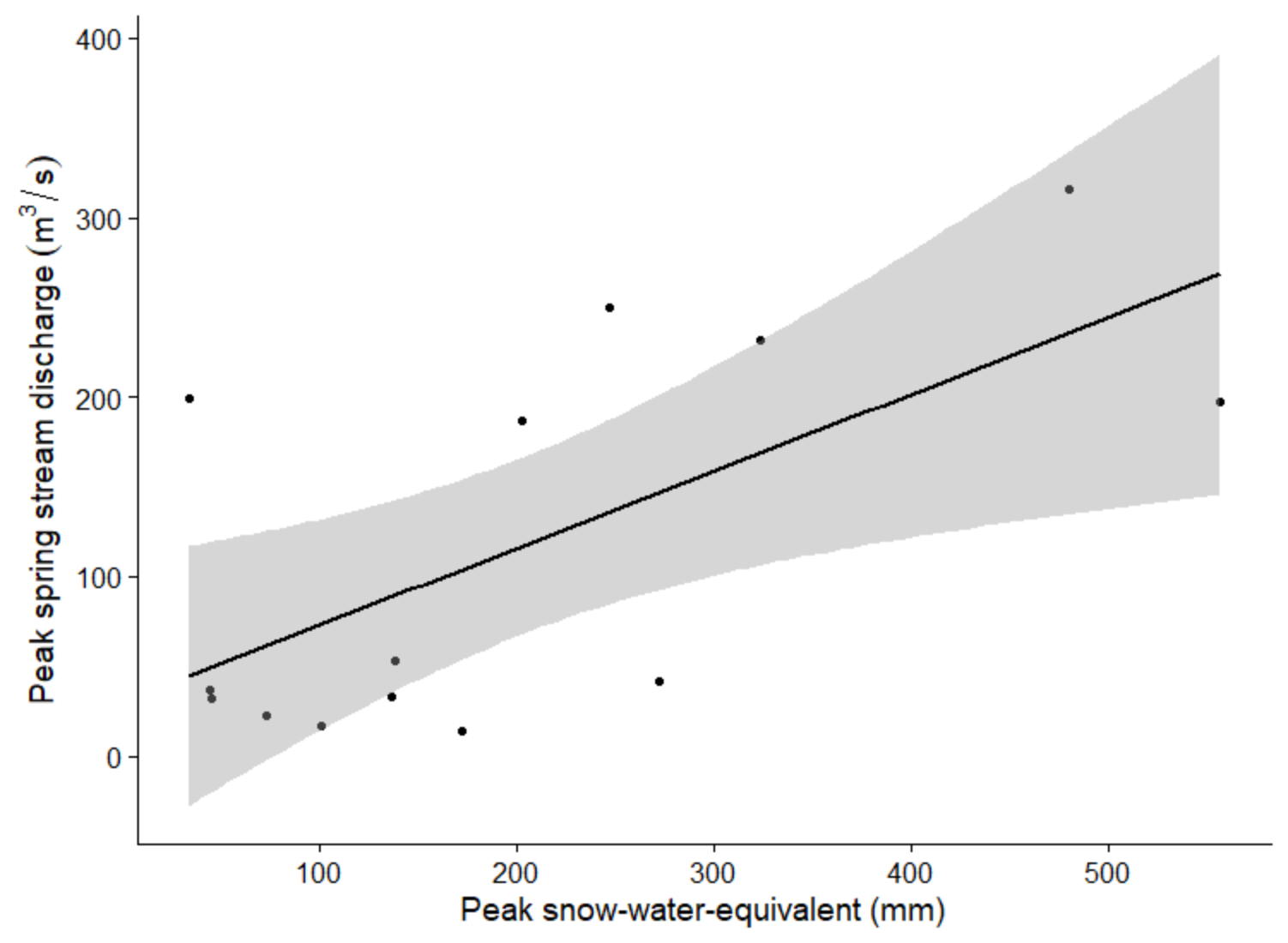


*Figure S2. Relationship between peak snow-water-equivalent, generated using a water balance model (Tercek et al. 2022), and peak spring Bright Angel Creek discharge (USGS gage 09403000 data).*

We used air temperature data provided in Tillman et al. (2020) to develop maximum stream temperature scenarios for BAC. Air temperature increases of up to 3.4°C were forecasted for the Grand Canyon region by the end of the century, and 2.8°C is projected by 2050 (Tillman et al. 2020). We used a subset of air temperature data for 2022 -2050 to match available years of data from Wheeler et al. (2021) for future Alternative Management Paradigms for Colorado River reservoirs. We used a published model developed to predict water temperature by location in BAC to convert maximum air temperatures to maximum annual water temperature when water is or is not diverted for human use from Roaring Springs (Bair et al. 2019):

*BAC_stream temp, subpop i._ = (3.615 – 1.710P) + 0.282D+ (0.340 + 0.085P) × A*,

where *P* = diversion pump on(1)/off(0), *D*=longitudinal distance from Roaring Springs, *A*=air temperature at Phantom Ranch (near the mouth of BAC). Some model calibration and assumptions were required to match maximum stream temperatures chosen to represent the baseline maximum temperature for the BACL, BACM, and BACU subpopulations from available NPS and USGS water temperature loggers placed along BAC. We assumed the diversion was constantly applied to represent reduced (~20%) future flows in BAC (Bair et al. 2019). Lacking future air temperature projections from across the range of BAC elevations, we generated % annual temperature increases from regional air temperature increases in Tillman et al. (2020) data (Fig. S3), and then applied those % increases to air temperature in the Bair et al. (2019) model for each subpopulation, starting with 35°C as a baseline air temperature (Table S4). Future temperatures for BAC subpopulations were included with annual discharge for climate changes scenarios as described above. R code to develop these temperature scenarios is included below.


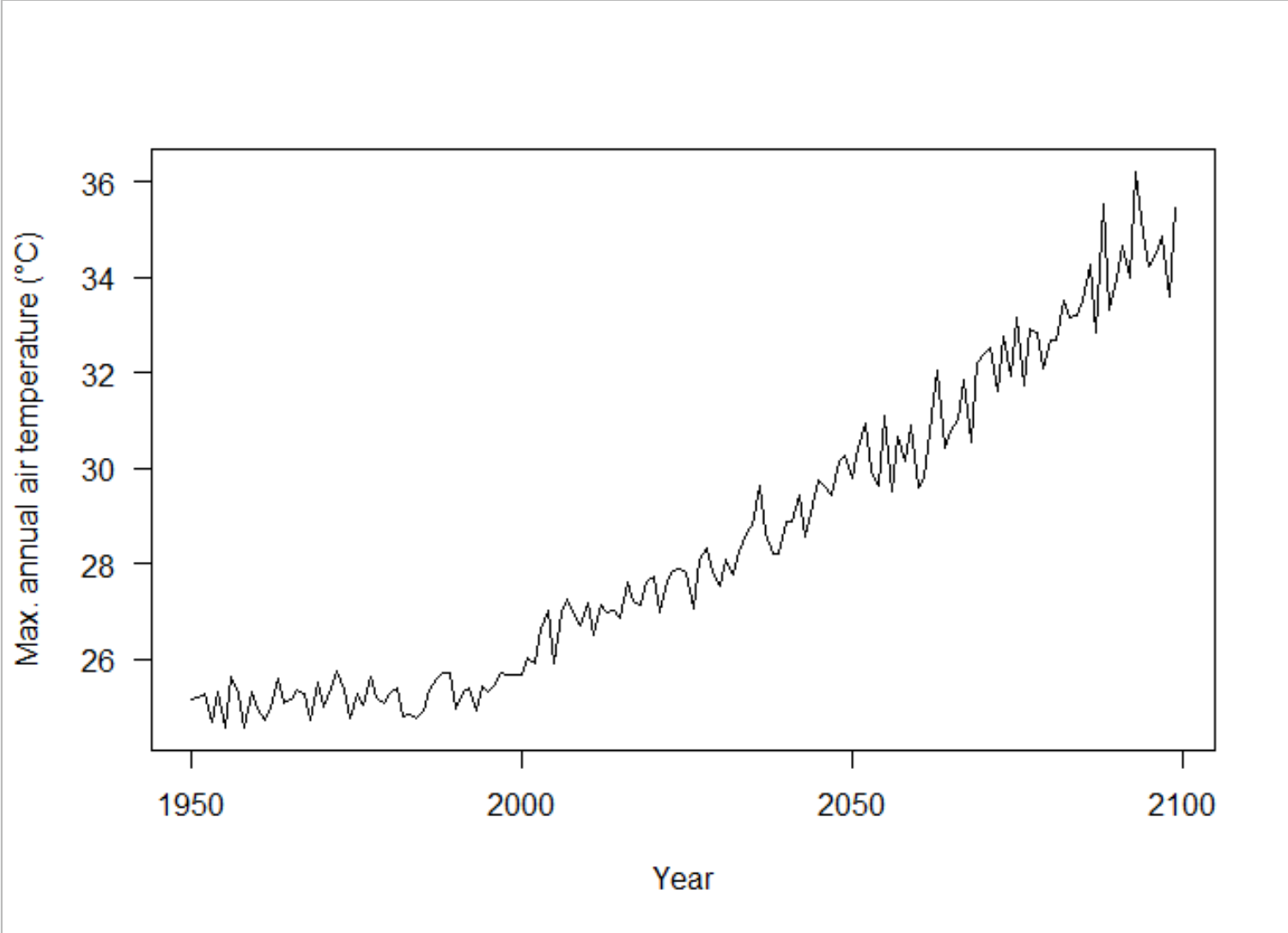


*Figure S3. Monthly max air temperatures using Tillman et al. (2020) data from USGS website (converted to average from 370 grids, then to Celsius). These data were subset from 2021-2050 for use in BAC water temp models.*

Table S4. Water temperature modeling results using maximum air temperature from Tillman et al. (2020)(MaxMnAnnTemp), and proportional increases (inc) applied to Phantom Ranch baseline air temperature (PRair; 35°C), which were used to generate subpopulation-specific proportional increases in water temperatures (right 3 columns) using the Bair et al. (2019) water temperature model from baselines of 16°C, 18°C, and 23°C for BACU, BACM, and BACL, respectively.

| yr | MaxMnAnnTemp | inc | PRair | BACUtemp | BACMtemp | BACLtemp | BACUtemp.adj | BACMtemp.adj | BACLtemp.adj |
| --- | --- | --- | --- | --- | --- | --- | --- | --- | --- |
| 2021 | 26.99 | 0.00 | 35.00 | 17.06 | 17.91 | 20.45 | 16.00 | 18.00 | 23.00 |
| 2022 | 27.65 | 0.66 | 35.66 | 17.34 | 18.19 | 20.73 | 16.26 | 18.28 | 23.31 |
| 2023 | 27.86 | 0.87 | 35.87 | 17.43 | 18.28 | 20.81 | 16.34 | 18.37 | 23.41 |
| 2024 | 27.87 | 0.88 | 35.88 | 17.44 | 18.28 | 20.82 | 16.35 | 18.38 | 23.42 |
| 2025 | 27.83 | 0.84 | 35.84 | 17.42 | 18.26 | 20.80 | 16.33 | 18.36 | 23.40 |
| 2026 | 27.05 | 0.06 | 35.06 | 17.09 | 17.93 | 20.47 | 16.02 | 18.02 | 23.03 |
| 2027 | 28.08 | 1.09 | 36.09 | 17.53 | 18.37 | 20.91 | 16.43 | 18.46 | 23.52 |
| 2028 | 28.31 | 1.32 | 36.32 | 17.62 | 18.47 | 21.01 | 16.52 | 18.56 | 23.63 |
| 2029 | 27.82 | 0.83 | 35.83 | 17.42 | 18.26 | 20.80 | 16.33 | 18.35 | 23.40 |
| 2030 | 27.53 | 0.54 | 35.54 | 17.29 | 18.14 | 20.67 | 16.21 | 18.23 | 23.25 |
| 2031 | 28.07 | 1.08 | 36.08 | 17.52 | 18.37 | 20.91 | 16.43 | 18.46 | 23.52 |
| 2032 | 27.76 | 0.77 | 35.77 | 17.39 | 18.23 | 20.77 | 16.30 | 18.33 | 23.36 |
| 2033 | 28.23 | 1.24 | 36.24 | 17.59 | 18.44 | 20.97 | 16.49 | 18.53 | 23.59 |
| 2034 | 28.66 | 1.67 | 36.67 | 17.77 | 18.62 | 21.16 | 16.66 | 18.71 | 23.80 |
| 2035 | 28.80 | 1.81 | 36.81 | 17.83 | 18.68 | 21.22 | 16.72 | 18.77 | 23.86 |
| 2036 | 29.65 | 2.66 | 37.66 | 18.19 | 19.04 | 21.58 | 17.06 | 19.13 | 24.27 |
| 2037 | 28.64 | 1.65 | 36.65 | 17.77 | 18.61 | 21.15 | 16.66 | 18.71 | 23.79 |
| 2038 | 28.19 | 1.20 | 36.20 | 17.57 | 18.42 | 20.96 | 16.48 | 18.51 | 23.57 |
| 2039 | 28.21 | 1.22 | 36.22 | 17.58 | 18.43 | 20.96 | 16.48 | 18.52 | 23.58 |
| 2040 | 28.87 | 1.88 | 36.88 | 17.86 | 18.71 | 21.24 | 16.75 | 18.80 | 23.89 |
| 2041 | 28.89 | 1.90 | 36.90 | 17.87 | 18.71 | 21.25 | 16.75 | 18.81 | 23.90 |
| 2042 | 29.42 | 2.43 | 37.43 | 18.09 | 18.94 | 21.48 | 16.97 | 19.04 | 24.16 |
| 2043 | 28.56 | 1.57 | 36.57 | 17.73 | 18.57 | 21.11 | 16.62 | 18.67 | 23.75 |
| 2044 | 29.24 | 2.25 | 37.25 | 18.02 | 18.86 | 21.40 | 16.89 | 18.96 | 24.07 |
| 2045 | 29.75 | 2.76 | 37.76 | 18.23 | 19.08 | 21.62 | 17.10 | 19.18 | 24.32 |
| 2046 | 29.59 | 2.60 | 37.60 | 18.17 | 19.01 | 21.55 | 17.04 | 19.11 | 24.24 |
| 2047 | 29.42 | 2.43 | 37.43 | 18.09 | 18.94 | 21.48 | 16.97 | 19.04 | 24.16 |
| 2048 | 30.15 | 3.16 | 38.16 | 18.40 | 19.25 | 21.79 | 17.26 | 19.35 | 24.51 |
| 2049 | 30.28 | 3.29 | 38.29 | 18.46 | 19.31 | 21.84 | 17.31 | 19.40 | 24.57 |
| 2050 | 29.80 | 2.81 | 37.81 | 18.25 | 19.10 | 21.64 | 17.12 | 19.20 | 24.34 |

*GCregionTemps<-read.csv("C:/Users/…/GC_area_TMEAN_monthly_MEAN_F.csv",header=TRUE)*

*library(frost)*

*GCregionTemps$cels <- convert.temperature(from="F",to="C",GCregionTemps$avgTemp)*

*GCregionmaxtemp<- ddply(GCregionTemps,.(yr), summarise,MaxMnAnnTemp=max(cels, na.rm=TRUE))*

*plot(MaxMnAnnTemp~yr,data=GCregionmaxtemp, type="l", las=1, xlab="Year",ylab="Max. annual air temperature (\u00B0C)")*

*GCregionmaxtemp2050<-subset(GCregionmaxtemp, yr>2020 &yr<2051)*

#calculate annual temp increase from baseline 26.99 air temp in 2021

*GCregionmaxtemp2050$inc<-GCregionmaxtemp2050$MaxMnAnnTemp-26.99*

#calc Phantom Ranch future air temp from baseline 35 from Bair et al.

*GCregionmaxtemp2050$PRair<-GCregionmaxtemp2050$inc+35*

#calc future water temp for each Subpopulation by adding to temp used in base model (with pump on)

*GCregionmaxtemp2050$BACUtemp<-(3.615-1.71*1)+(0.282*1)+((0.34+0.085*1)*GCregionmaxtemp2050$PRair)*

*GCregionmaxtemp2050$BACMtemp<-(3.615-1.71*1)+(0.282*4)+((0.34+0.085*1)*GCregionmaxtemp2050$PRair)*

*GCregionmaxtemp2050$BACLtemp<-(3.615-1.71*1)+(0.282*13)+((0.34+0.085*1)*GCregionmaxtemp2050$PRair)*

*Perturbation - sensitivity analysis –* Due to uncertainty in relationships between North Kaibab Plateau peak SWE and BAC discharge, and a large number of assumptions related to climate change scenarios, we conducted a sensitivity analysis to understand how varying peak SWE thresholds representing extreme high and low spring discharge years would affect age-0 brown trout survival. We increased or decreased (10% and 20%) the assumed peak SWE values that would represent high and low spring peak discharge (5.7 and 0.57 m^3^/s) from baselines of >220 or <30 mm SWE. We used the CR resample 2000 baseline model (assumes status quo reservoir management) for all sensitivity analysis scenarios. Results of sensitivity analysis, comparing minimum population sizes (*N_min_*) between baseline wet (INMCM.4) and dry (BNU.ESM) models, are shown in Table S5. *N_min_* values generally deviated little from baseline models when thresholds were changed, and BACU, was most sensitive to changes in thresholds (-2.9% to 14.6%). Observed predicted changes in subpopulation *N_min_* averaged -2.5% to 4.3% from the baseline.

Table. S5. Results of climate change scenario sensitivity analysis, involving adjustment of peak snow-water-equivalent and peak spring Bright Angel Creek discharge relationships so that dry and wet scenario thresholds are +/-10% or +/- 20% of the baseline. The upper section of the table shows the number of occurrences of high or low spring peak flows (above and below optimum range for brown trout age-0 survival) with changes in thresholds, and the lower portion of the table represents % changes in minimum population abundance for the metapopulation and each subpopulation, with changes in threholds.

|  | Climate- CRbasewet | Wet, threshold -10% | Wet, threshold +10% | Wet, threshold -20% | Wet, threshold +20% |  | Climate-CRbasedry | Dry, threshold -10% | Dry, threshold +10% | Dry, threshold -20% | Dry, threshold +20% |
| --- | --- | --- | --- | --- | --- | --- | --- | --- | --- | --- | --- |
| High and low spring peak runoff frequency (number/30 years) | | | | | | | | | | | |
| Low flows | 2 | 1 | 3 | 1 | 3 |  | 6 | 5 | 6 | 4 | 6 |
| High flows | 6 | 6 | 4 | 7 | 1 |  | 4 | 4 | 3 | 5 | 3 |
| Total disturbances | 8 | 7 | 7 | 8 | 4 |  | 10 | 9 | 9 | 9 | 9 |
| Minimum metapopulation and subpopulation abundances and % change from baseline model | | | | | | | | | | | |
| Meta | 10771 | 1.42% | -2.50% | 1.78% | 0.87% |  | 9422 | 1.58% | 1.05% | 2.51% | 2.66% |
| BACL | 374 | 3.61% | -1.63% | 3.86% | 0.00% |  | 335 | 1.18% | -0.30% | 4.83% | 2.90% |
| BACM | 1575 | 3.85% | -9.38% | 4.95% | 2.05% |  | 959 | 2.74% | 5.70% | 4.67% | 6.71% |
| BACU | 1180 | -2.88% | 0.84% | -9.87% | 14.62% |  | 683 | 8.45% | 12.21% | 3.94% | 14.30% |
| CR | 7505 | 1.20% | 0.07% | 0.91% | -0.07% |  | 7416 | 1.12% | -0.31% | 2.37% | 1.25% |

***Appendix S4: Validation of the PVA results using observed vs simulated trends in Bright Angel Ceek abundance.***

We used data from long-term suppression activities in BAC (Healy et al., 2020, 2022) for estimating size- (age) specific electrofishing capture probability ($\hat{p}$) to be used in BAC suppression scenarios. Initial model tests using estimates of $\hat{p}$ taken from Healy et al. (2022) caused the BAC subpopulations to reach quasi-extinction (QE) at a faster rate than observed in BAC (Healy et al. 2020). Thus, we made adjustments until declines approximated observed declines in BAC (Fig. S4). Results of analysis of environmental and spatial variation in $\hat{p}$ (Healy et al., 2022) found 15% and 49% reductions in $\hat{p}$ for BACM and BACU subpopulations relative to BACL, and we adjusted subpopulation $\hat{p}$ accordingly (Table S6). Comparing observed and simulated abundance over 7 years of suppression in BAC suggested similar outcomes could be represented by our PVA, although observed declines were greater than simulated in BACL, which is likely due to immigration from the CR subpopulation. We also note that 2020-21 abundance increased substantially, likely due to optimum flows for age-0 recruitment, corresponding to year 8 in Fig. S4.

Table S6. Cumulative capture probability ($\hat{p}$) values used for BAC brown trout subpopulation suppression scenarios for 2-4 electrofishing passes.

|  | 2-pass $\hat{p}$ | 3-pass $\hat{p}$ | 4-pass $\hat{p}$ |
| --- | --- | --- | --- |
| Bright Angel Creek - lower subpopulation |  |  |  |
| Age0 | 0.48 | 0.58 | 0.78 |
| Juvenile | 0.61 | 0.74 | 0.89 |
| SmallAdult | 0.65 | 0.79 | 0.93 |
| LargeAdult | 0.70 | 0.85 | 0.96 |
| Bright Angel Creek - middle subpopulation |  |  |  |
| Age0 | 0.38 | 0.49 | 0.68 |
| Juvenile | 0.49 | 0.63 | 0.81 |
| SmallAdult | 0.52 | 0.67 | 0.84 |
| LargeAdult | 0.56 | 0.72 | 0.87 |
| Bright Angel Creek - upper subpopulation |  |  |  |
| Age0 | 0.20 | 0.30 | 0.44 |
| Juvenile | 0.26 | 0.38 | 0.54 |
| SmallAdult | 0.28 | 0.40 | 0.56 |
| LargeAdult | 0.30 | 0.43 | 0.60 |


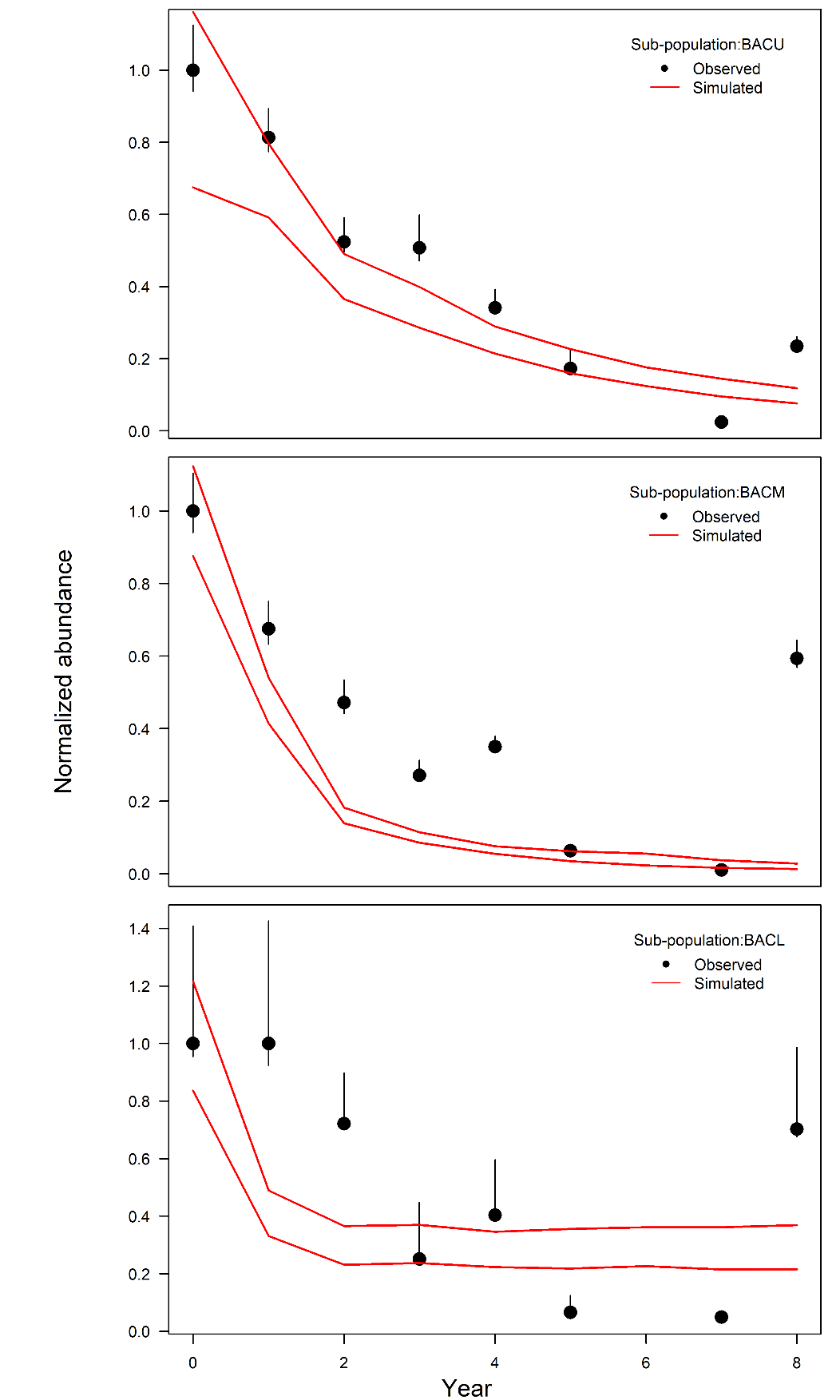


*Figure S4. Validation plots of observed for reaches 1, 3, and 5 through spring 2021 (Healy et al. 2020, NPS unpublished 2020-21 abundance data) compared to simulated trends in normalized abundance (abundance trends normalized to % of carrying capacity) for BACL, BACM, BACU subpopulations.*

**LITERATURE CITED**

Bair RT, Tobin BW, Healy BD, Spangenberg CE, Childres HK, Schenk ER. 2019. Modeling temperature regime and physical habitat impacts from restored streamflow. Environmental Management **63**:718–731.

Bates D, Maechler M, Bolker B, Walker S. 2015. Fitting linear mixed effects models using lme4. Journal of Statistical Software **67**:1–48.

Birnie-Gauvin K, Thorstad EB, Aarestrup K. 2019. Overlooked aspects of the Salmo salar and Salmo trutta lifecycles. Reviews in Fish Biology and Fisheries **29**:749–766. Springer International Publishing. Available from https://doi.org/10.1007/s11160-019-09575-x.

Budy P, Thiede GP, McHugh P, Hansen ES, Wood J. 2008. Exploring the relative influence of biotic interactions and environmental conditions on the abundance and distribution of exotic brown trout (Salmo trutta) in a high mountain stream. Ecology of Freshwater Fish **17**:554–566.

Burnham KP, Anderson DR. 2002. Model selection and multimodel inference: a practical information-theoretic approach2nd editio. Springer-Verlag New York, Inc., New York.

Ciepiela LR, Walters AW. 2019. Life-history variation of two inland salmonids revealed through otolith microchemistry analysis. Canadian Journal of Fisheries and Aquatic Sciences **76**:1971–1981.

Cucherousset J, Ombredane D, Charles K, Marchand F, Baglinière JL. 2005. A continuum of life history tactics in a brown trout (Salmo trutta) population. Canadian Journal of Fisheries and Aquatic Sciences **62**:1600–1610.

Eisenhauer ZJ, Christman PM, Matte J-M, Ardren WR, Fraser DJ, Grant JWA. 2020. Revisiting the restricted movement paradigm: the dispersal of Atlantic salmon fry from artificial redds. Canadian Journal of Fisheries and Aquatic Sciences **11**:1–11.

Forseth T, Larsson S, Jensen AJ, Jonsson B, Näslund I, Berglund I. 2009. Thermal growth performance of juvenile brown trout Salmo trutta: No support for thermal adaptation hypotheses. Journal of Fish Biology **74**:133–149.

Goodwin JCA, Andrew King R, Iwan Jones J, Ibbotson A, Stevens JR. 2016. A small number of anadromous females drive reproduction in a brown trout (Salmo trutta) population in an English chalk stream. Freshwater Biology **61**:1075–1089.

Grossman GD, Carline RF, Wagner T. 2017. Population dynamics of brown trout (Salmo trutta) in Spruce Creek Pennsylvania: A quarter-century perspective. Freshwater Biology **62**:1143–1154.

Hammond JC, Saavedra FA, Kampf SK. 2018. How Does Snow Persistence Relate to Annual Streamflow in Mountain Watersheds of the Western U.S. With Wet Maritime and Dry Continental Climates? Water Resources Research **54**:2605–2623.

Healy BD, Moore JF, Pine WE. 2022. Monitoring and managing fishes that are invisible and keep moving around: influences of an invasive species and environmental factors on capture probability. North American Journal of Fisheries Management **43**:396–409.

Healy BD, Schelly RC, Yackulic CB, Smith ECO, Budy P. 2020. Remarkable response of native fishes to invasive trout suppression varies with trout density, temperature, and annual hydrology. Canadian Journal of Fisheries and Aquatic Sciences **77**:1446–1462. Available from https://doi.org/10.1139/cjfas-2020-0028.

Healy BD, Yackulic CB, Schelly RC. 2023 Impeding access to tributary spawning habitat and releasing experimental fall-timed floods increases brown trout immigration into a dam's tailwater. Canadian Journal of Fisheries and Aquatic Sciences. *In press.*

Hughes NF, Grand TC. 2000. Physiological ecology meets the ideal-free distribution: predicting the distribution of size-structured fish populations across temperature gradients. Environmental Biology of Fishes **59**:285–298.

Ji D et al. 2014. Description and basic evaluation of Beijing Normal University Earth System Model (BNU-ESM) version 1. Geoscientific Model Development **7**:2039–2064.

Jonsson B, Jonsson N. 2009. A review of the likely effects of climate change on anadromous Atlantic salmon Salmo salar and brown trout Salmo trutta , with particular reference to water temperature and flow. Journal of Fish Biology **75**:2381–2447. Available from http://doi.wiley.com/10.1111/j.1095-8649.2009.02380.x.

Jorgensen J, Berg S. 1991. Stocking experiments with 0 + and 1 + trout parr, Salmo trutta L., of wild and hatchery origin: 2. Post‐stocking movements. Journal of Fish Biology **39**:171–180.

Korman J, Kaplinski M, Melis TS. 2011. Effects of fluctuating flows and a controlled flood on incubation success and early survival rates and growth of age-0 rainbow trout in a large regulated river. Transactions of the American Fisheries Society **140**:487–505.

Lawrence DJ, Runyon AN, Gross JE, Schuurman GW, Miller BW. 2021. Divergent , plausible , and relevant climate futures for near- and long-term resource planning. Climate Change **167**:1–20. Climatic Change.

Lobón-Cerviá J. 2009. Why, when and how do fish populations decline, collapse and recover? the example of brown trout (Salmo trutta) in Rio Chaballos (northwestern Spain). Freshwater Biology **54**:1149–1162.

Lobón-Cerviá J, Rasmussen GH, Mortensen E. 2018. Discharge-dependent recruitment in stream-spawning brown trout. Pages 297–318 in J. Lobón-Cerviá and N. Sanz, editors. Brown Trout: Life History, Ecology and Management, 1st edition. John Wiley & Sons Ltd. Available from http://doi.wiley.com/10.1002/9781119268352.ch13.

Macdonald PDM, Pitcher TJ. 1979. Age-groups from size-frequency data: a versatile and efficient method of analyzing distribution mixtures. Journal of the Fisheries Research Board of Canada **36**:987–1001. Available from http://www.nrcresearchpress.com/doi/10.1139/f79-137.

Milner NJ, Elliott JM, Armstrong JD, Gardiner R, Welton JS, Ladle M. 2003. The natural control of salmon and trout populations in streams. Fisheries Research **62**:111–125.

Murphy BP, Walsworth TE, Belmont P, Conner MM, Budy P. 2020. Dynamic habitat disturbance and ecological resilience ( DyHDER ): modeling population responses to habitat condition. Ecosphere **11**.

R Core Team. 2019. R: A language and environment for statistical computing. R foundation for statistical computing, Vienna, Austria.

Radinger J, Wolter C. 2014. Patterns and predictors of fish dispersal in rivers. Fish and Fisheries **15**:456–473.

Railsback SF, Rose KA. 1999. Bioenergetics Modeling of Stream Trout Growth: Temperature and Food Consumption Effects. Transactions of the American Fisheries Society **128**:241–256.

Richter BD, Baumgartner J V, Powell J, Braun DP. 1996. A method for assessing hydrologic alteration within ecosystems. Conservation Biology **10**:1163–1174.

Rogowski DL, Boyer JK. 2019. Colorado River fish monitoring in Grand Canyon , Arizona — 2018 annual report. Arizona Game and Fish Department, submitted to the Grand Canyon Monitoring and Research Center. 47 pages, Flagstaff, Arizona.

Rosenfeld JS. 2017. Developing flow–ecology relationships: Implications of nonlinear biological responses for water management. Freshwater Biology **62**:1305–1324.

Runge MC, Yackulic CB, Bair LS, Kennedy TA, Valdez RA, Ellsworth C, Kershner JL, Scott Rogers R, Trammell MA, Young KL. 2018. Brown trout in the Lees Ferry reach of the Colorado River—evaluation of causal hypotheses and potential interventions. Flagstaff, Arizona.

Smialek N, Pander J, Geist J. 2021. Environmental threats and conservation implications for Atlantic salmon and brown trout during their critical freshwater phases of spawning, egg development and juvenile emergence. Fisheries Management and Ecology:1–31.

Stewart IT. 2009. Changes in snowpack and snowmelt runoff for key mountain regions. Hydrological Processes **23**:78–94. Available from http://jamsb.austms.org.au/courses/CSC2408/semester3/resources/ldp/abs-guide.pdf.

Syrjänen JT, Vainikka A, Louhi P, Huusko A, Orell P, Vehanen T. 2017. History, conservation and management of adfluvial brown trout stocks in Finland. Pages 697–733 in J. Lobón‐Cerviá and N. Sans, editors. Brown Trout: Life History, Ecology and ManagementFirst edit. John Wiley & Sons Ltd.

Syslo JM, Brenden TO, Guy CS, Koel TM, Bigelow PE, Doepke PD, Arnold JL, Ertel BD. 2020. Could ecological release buffer suppression efforts for non-native lake trout (Salvelinus namaycush) in Yellowstone Lake, Yellowstone National Park? Canadian Journal of Fisheries and Aquatic Sciences **77**:1010–1025.

Tercek MT, Thoma D, Gross JE, Sherrill K, Kagone S, Senay G. 2021. Historical changes in plant water use and need in the continental United States. PLoS ONE **16**.

Tillman FD, Gangopadhyay S, Pruitt T. 2020. Recent and projected precipitation and temperature changes in the Grand Canyon area with implications for groundwater resources. Scientific Reports:1–11. Nature Publishing Group UK.

U.S. Geological Survey [USGS], 2022, USGS water data for the Nation: U.S. Geological Survey National Water Information System database, accessed April 1, 2022, at https://doi.org/10.5066/F7P55KJN.

Vatland S, Caudron A. 2015. Movement and early survival of age-0 brown trout. Freshwater Biology **60**:1252–1262.

Vøllestad LA, Serbezov D, Bass A, Bernatchez L, Olsen EM, Taugbøl A. 2012. Small-scale dispersal and population structure in stream-living brown trout (Salmo trutta) inferred by mark-recapture, pedigree reconstruction, and population genetics. Canadian Journal of Fisheries and Aquatic Sciences **69**:1513–1524.

Volodin EM, Dianskii NA, Gusev A V. 2010. Simulating present-day climate with the INMCM4.0 coupled model of the atmospheric and oceanic general circulations. Izvestiya - Atmospheric and Ocean Physics **46**:414–431.

Wheeler K et al. 2021. Alternative Management Paradigms for the Future of the Colorado and Green Rivers. Center for Colorado River Studies, Quinney College of Natural Resources, Utah State University White Paper No. 6. https://qcnr.usu.edu/coloradoriver/files/WhitePaper6.pdf., Logan, Utah.

Whiting DP, Paukert CP, Healy BD, Spurgeon JJ. 2014. Macroinvertebrate prey availability and food web dynamics of nonnative trout in a Colorado River tributary, Grand Canyon. Freshwater Science **33**:872–884.

Yackulic CB, Dodrill M, Dzul M, Sanderlin JS, Reid JA. 2020. A need for speed in Bayesian population models: a practical guide to marginalizing and recovering discrete latent states. Ecological Applications **30**:1–19.

Yard MD, Coggins LG, Baxter C V., Bennett GE, Korman J. 2011. Trout piscivory in the Colorado River, Grand Canyon: effects of turbidity, temperature, and fish prey availability. Transactions of the American Fisheries Society **140**:471–486.
